# Research on the Composition and Casting Technology of Bronze Arrowheads Unearthed from the Ruins of the Imperial City of the Minyue Kingdom

**DOI:** 10.3390/ma18020402

**Published:** 2025-01-16

**Authors:** Lei Zhang, Yile Chen, Liang Zheng, Ruyi Zheng

**Affiliations:** 1 School of Civil Engineering and Architecture, Wuyi University, No. 358 Baihua Road, Wuyishan 354300, China; zhanglei@wuyiu.edu.cn (L.Z.); zry081600@163.com (R.Z.); 2 Faculty of Humanities and Arts, Macau University of Science and Technology, Avenida Wai Long, Tapai, Macau 999078, China; me@zhengliang.org; 3 Heritage Conservation Laboratory, Macau University of Science and Technology, Avenida Wai Long, Tapai, Macau 999078, China

**Keywords:** bronze arrowheads, corrosion phenomenon, bronze heritage, metallographic analysis, copper–tin alloy, Minyue Kingdom, component analysis

## Abstract

The ruins of the Imperial City of the Minyue Kingdom were an important site of the Minyue Kingdom during the Han Dynasty. Characteristic bronze arrowheads unearthed from the East Gate, with their exquisite craftsmanship, provide important physical evidence for studying ancient bronze casting technology and the military activities of that time. However, there is still a lack of systematic research on the alloy composition, casting process, and chemical stability of these arrowheads in long-term burial environments. The bronze arrowheads that were found in the East Gate warehouse are the subject of this study. Metallographic analysis, scanning electron microscopy (SEM), and energy-dispersive spectroscopy (EDS) were used to carefully examine their composition and microstructure, as well as the casting process characteristics. The findings reveal the following: (1) The East Gate bronze arrowheads primarily consist of copper–tin binary alloys, and certain samples exhibit a lead (Pb) content of up to 11.19%, potentially due to element addition during casting or element migration in the burial environment. (2) The metallographic structure shows that the sample matrix has a typical α-dendrite structure, indicating that a high-temperature casting process was used, and then a certain surface treatment was performed to enhance corrosion resistance. (3) Under a scanning electron microscope, it was observed that a three-layer structure was formed on the surface of the arrowhead, including a fully mineralized layer, an intermediate transition layer, and the original core tissue. (4) The detection of molybdenum (Mo) in some samples suggests a close relationship between the complexity of the buried soil environment and human activities. (5) By comparing the microstructure and corrosion degree of the longitudinal section and the cross-section, it was found that the longitudinal section has a stronger corrosion resistance due to its denser structure. Comprehensive analysis shows that the technical details of the bronze arrowheads unearthed from the Minyue Imperial City in terms of material selection, casting process, and later use reflect the outstanding achievements of the Minyue Kingdom in the field of bronze manufacturing in the Han Dynasty.

## 1. Introduction

### 1.1. Research Background and Purpose

The production technology and usage of bronze artifacts, material carriers important for the development of early human civilization, not only reflect the scientific and technological levels and cultural characteristics of ancient society but also provide valuable physical materials for studying the economic, military, and political structure of ancient society. The protection and research of metal heritage has received extensive attention in the fields of archeology and materials science in recent years [1,2,3]. In particular, for bronze artifacts buried underground, their surfaces and internal materials undergo irreversible changes in complex physical and chemical environments due to various phenomena, such as the presence of aggressive anions and microbial infestation. Generally speaking, the environment in which bronze artifacts are buried is oxygen-deficient for a long period of time. Once unearthed, the bronze artifacts are exposed to air for a prolonged period and over a wider area. In this oxygen-rich environment, the probability of bronze artifacts rusting and oxidizing is relatively high. The air contains a large amount of water vapor, CO_2_, O_2_, etc. Large amounts of these gases can be absorbed by the loose rust layer on the surfaces of recently discovered bronze artifacts. This directly contributes to the corrosion process and creates powdery rust on the surface of the bronze artifact matrix. These changes not only affect the preservation status of the artifacts but also record their usage history and environmental changes to a certain extent [4]. Thus, performing thorough studies on the types of materials used, the casting process, and how bronze artifacts rust will help us to understand how ancient technology changed over time [5]. This will also help researchers to better use scientific analysis methods in the future to promote the protection of bronze cultural heritage.

The analysis methods for ancient bronzes in existing academic research primarily focus on two areas: the restoration of the material composition and manufacturing process, and the relationship between the burial environment and the preservation state of cultural relics. The former uses metallographic microscopic analysis, scanning electron microscopy (SEM), energy-dispersive spectrometer (EDS) analysis, and other technical means to explore the alloy ratio, casting process, and microstructural characteristics, providing a scientific basis for understanding the production process of bronzes [6,7,8]; the latter focuses more on the impact of the burial environment on the stability of cultural relic materials, revealing the driving role of environmental factors, such as the soil pH and redox reactions, on corrosion mechanisms [9,10,11]. These studies have not only deepened our understanding of ancient metal processing technology but have also promoted the advancement of metal cultural relic protection technology.

The bronze ritual vessels and weapon manufacturing technology of the Shang and Zhou dynasties (approximately 1600 BC to 256 BC) have attracted much academic attention. For instance, ref. [12] conducted a detailed analysis on bronze arrowheads unearthed from the Yuwan cemetery in Hubei Province, scrutinizing the production process of Chu weapons and its correlation with the source of the materials. Similarly, ref. [13] employed metal isotope analysis methods to investigate the origin of the raw materials at the bronze weapon casting site of the Zhu State in Shandong Province, providing crucial evidence for the movement of metal resources during the Shang and Zhou dynasties. However, research on the weapon manufacturing technology of local governments in the Han Dynasty (202 BC to 220 AD) is still limited. Studies on metal arrowheads unearthed from Western Han tombs near the Han Dynasty Chang’an city ruins have revealed the manufacturing and corrosion characteristics of metal weapons in the core area of the Han Dynasty [14], but there is still a lack of in-depth analysis of the bronze weapon manufacturing and use of technology by local governments in the southeast region of the Han Dynasty, especially the Minyue Kingdom.

The bronze arrowhead manufacturing technology of the Minyue Kingdom, an important local regime in the southeastern region of the Han Dynasty, may have shown characteristics that were completely different from those in the Central Plains. The archeological discoveries of the ruins of the Imperial City of the Minyue Kingdom specifically reflect these characteristics [15]. The Fujian Provincial Cultural Relics Administration conducted an archeological survey in 1958, and subsequent excavations of the East Gate site revealed the unique technical traditions of this region in the production of military equipment. The East Gate warehouse unearthed a large number of clustered bronze arrowheads, exquisitely made with obvious regional characteristics. For instance, these arrowheads not only exhibited the shape of sharp blades capable of penetrating enemy armor or shields but also underwent fine grinding and polishing to guarantee flight stability and accuracy. Additionally, some arrowheads featured surface-engraved patterns or symbols, potentially representing cultural or military symbols. This specific physical evidence shows that the Minyue Kingdom may have adopted a combination of rough casting and forging in the casting process to meet the high standards of weapons in actual combat.

In addition, the unearthed morphology and distribution of bronze artifacts also suggest the unique storage and use habits of the region [16]. For instance, the East Gate site’s warehouse primarily distributes arrowheads in clusters, suggesting a systematic storage method to enable army calls at any time. Meanwhile, the scattered distribution in other areas reveals variations in their condition during use or disposal on the battlefield. Combining metallographic analysis with scanning electron microscopy observation, this study further explored the characteristics of these bronze arrowheads in terms of microstructure, material composition, and casting process, and attempted to restore the technical process and organizational form of the Minyue Kingdom in the production of military equipment, thereby providing a scientific basis for understanding the technical characteristics of Han Dynasty bronze weapons in the southeast region and their connection with the Central Plains region, and laying the foundation for a broader regional technology comparative study.

Therefore, this study took the bronze arrowheads unearthed from the ruins of the Imperial City of the Minyue Kingdom as the research object. By combining metallographic analysis with SEM-EDS technology, this study attempted to technically restore the manufacturing process of the bronze arrowheads from the ruins of the Imperial City of the Minyue Kingdom and explore the relationship between material properties and their military applications. This study focused on three key issues: (1) What are the alloy composition and microstructural characteristics of the bronze arrowheads unearthed from the ruins of the Imperial City of the Minyue Kingdom? (2) What are the specific casting and processing technologies used to manufacture these arrowheads? (3) What effect does the burial environment have on the preservation state and material stability of bronze arrowheads?

### 1.2. Literature Review

The ruins of the Imperial City of the Minyue Kingdom are located in Wuyishan City, Fujian Province. It is one of the best-preserved Han Dynasty princely cities in southern China and has important historical, cultural, and economic value [15,17]. Experts and scholars at home and abroad highly praised the site in December 1999, listing it as a world cultural heritage site. As a world cultural heritage site, the Imperial City of the Minyue Kingdom not only shows the urban layout and architectural style of the local government of the Han Dynasty but also reflects the political, economic, and cultural characteristics of the southeast region of this dynasty.

#### 1.2.1. Related Research on Cultural Heritage in Fujian

In recent years, with the emphasis on cultural heritage protection and research, the academic community has made significant progress in exploring the spatial distribution of cultural heritage and its relationship with the natural and cultural environment. Previously, researchers in Fujian looked at how cultural heritage is spread out and what factors affect it. They used geographic information system (GIS) spatial analysis technology to show how cultural heritage is spread out in time and space and how it interacts with the natural and human environment [18]. Research points out that the distribution of cultural heritage in Fujian shows significant agglomeration, and its core distribution area has gradually shifted from the upstream areas to the southeastern coastal areas with the changing historical periods. The type of heritage has also transitioned from early ancient ruins to ancient buildings from the Ming and Qing Dynasties. In modern times, the heritage primarily consists of the sites of significant historical events and representative buildings. The distribution characteristics of cultural heritage in northern Fujian further highlight the close connection between Minyue culture and the natural environment [19]. The research indicates that the the cultural heritage of this areas is primarily distributed in lowland valleys and riverbanks below 400 m above sea level, exhibiting a significant distribution pattern along the river. Social and cultural elements, like the Minyue culture, ancient tea ceremony, and porcelain culture, closely relate to this distribution. Specifically, the Minyue period’s transportation network demonstrates close links with the period’s material cultural heritage, offering crucial insights into the mechanism of cultural heritage formation in this region. In the specific research on the Minyue Kingdom, researchers used the “Least-Cost Path” (LCP) model to reconstruct the transportation and cultural heritage network system [20].

#### 1.2.2. Focus on Ancient Chinese Bronze Arrowheads

The study of ancient bronzes has made some progress. For example, the analysis of bronze arrowheads from Chu State revealed their production technology and material sources, providing a new perspective for understanding the bronze smelting technology of the state [12]. At the same time, the study of bronze weapons in Xichuan has further deepened the understanding of the production process of early Chinese bronzes and provided a scientific basis for the protection and restoration of related cultural relics [21]. Researchers also looked at bronzes from the Warring States period in Pujiang, Chengdu. They looked at their metallurgical features and how they corroded to determine their material properties and how well they were preserved [22]. In terms of cultural exchange and technological integration, the study of bronze daggers unearthed from the Shuangyuan Village site has explored the interaction and technological sharing between different cultures in the Eastern Zhou Dynasty [23]. At the same time, the study of copper alloy production in the Chengdu area during the Warring States period has further revealed the characteristics of regional bronze smelting technology, providing key clues for understanding local resource utilization and social and technological development [24]. Scholars from Wuhan University also analyzed the corrosion morphology of bronze arrowheads from the Warring States period in the middle reaches of the Yangtze River. They found that, due to different degrees of corrosion, the organizational structure of the bronze arrowheads can be divided into three characteristic layers: a completely mineralized layer, a transition layer, and the original material in the core. Among them, the completely mineralized layer, whose main component is oxide, has the loosest structure and the lowest hardness [25,26,27,28].

#### 1.2.3. Technology and Methods of Analyzing Bronze Artifacts

In addition, there are different methods and techniques for analyzing and studying bronze heritage to further explore its composition and the mechanisms of layers’ corrosion. Iranian researchers used optical microscopy, scanning electron microscopy–energy-dispersive X-ray spectroscopy, and X-ray diffraction methods to study the corrosion layers of bronze artifacts from the Sangtarashan Iron Age site, western Iran. They found external corrosion products that have been identified as basic copper carbonates, malachite, and azurite [29]. Other researchers, from Portugal, used EDXRF, micro-EDXRF, SEM-EDS analysis, and metallographic examinations for artifacts dating to the end of the second millennium BC from Crasto de São Romão in Central Portugal. They stressed the importance of using multi-analytical methods in the field of cultural heritage, especially in the identification of ancient gilding techniques and bronze metallurgy. Therefore, traditional EDXRF analysis proved to be an excellent primary tool for studying large areas without surface treatment [30]. Using the same methodology, they also reviewed the development of metallurgy in Portugal over the first three thousand years after its emergence on the Iberian Peninsula [31]. Other scholars have used scanning electron microscopy and energy-dispersive spectrometry (scanning electron microscopy (SEM)–EDS) and optical microscopy techniques to analyze the structure and composition of some fragments of bronze belts and a bowl discovered from southwestern Armenia, in the Yegheghnadzor archeological site, from between 7 and 6 BCE in the Urartian period [32]. In addition to scanning electron microscopy + energy-dispersive spectrometry (SEM + EDS), X-ray diffraction (XRD), and optical microscopy (OM), the scholars G.M. Ingo and others innovatively used the combined technology of glow discharge optical emission spectrometry (GDOES) to study the corrosion products on the archeological leaded bronze artifacts used by the Punics and Romans [33]. A Chinese scholar named Liu Meijuan conducted a full study of the small bronze pieces found in the tomb of Wang Dahu from the Spring and Autumn and Warring States periods in Pengyang, Ningxia. She achieved this by using a handheld X-ray fluorescence spectrometer for non-destructive analysis, looking at the structures of the metals, using a scanning electron microscope to analyze the energy spectrum, and watching the production process [34]. This provided new scientific analysis data for the study and restoration of the development of bronze technology during the Spring and Autumn period and the Warring States period in Ningxia, China. Similarly, the scholar Zhang Pengyu used three-dimensional video microscopy, scanning electron microscopy, micro-area energy spectrum analysis, Raman analysis, metallographic analysis, and other analysis methods to conduct scientific detection and analysis on the base and rust products of a Warring States bronze sword in the Zhangqiu Museum in Shandong Province [35]. It can be seen that metallographic analysis and SEM–EDS analysis are basic methods and techniques for analyzing bronze cultural heritage.

In summary, these studies have collectively enhanced our understanding of ancient Chinese bronze manufacturing technology, cultural interactions, and regional differences. However, there is still a gap in the study of bronze artifacts specifically targeting the Minyue Kingdom in southeastern China. To fill this gap, this study aimed to further explore the material composition, casting process, and preservation mechanism of bronze arrowheads in combination with archeological discoveries from the ruins of the Imperial City of the Minyue Kingdom.

## 2. Materials and Methods

### 2.1. Study Area

**Figure 1:**
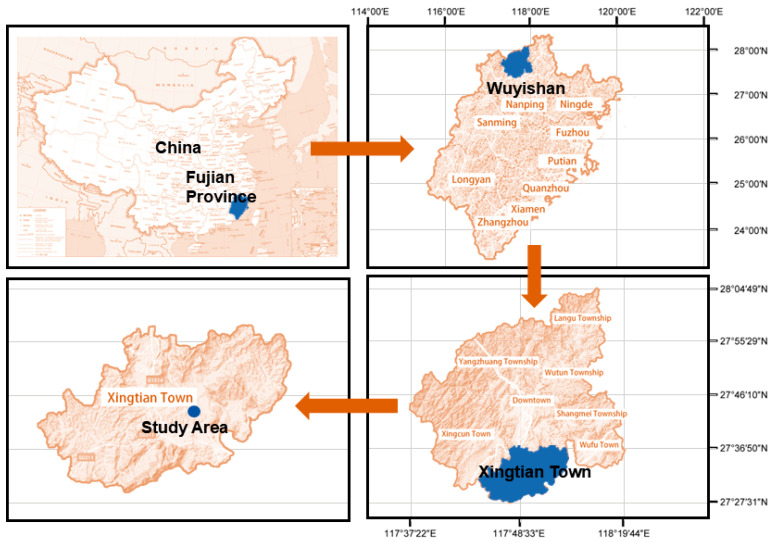
Location analysis (image source: drawn by the authors).

The bronze arrowheads in this study were unearthed from the ruins of the Imperial City of the Minyue Kingdom (also known as the Han Dynasty Ruins in Chengcun Village of Wuyishan City), which is located in Xingtian Town, Wuyishan City, Fujian Province, China (Figure 1). The early Western Han Dynasty built it on the southern foot of Wuyi Mountain, covering a total area of 480,000 km^2^. Wuzhu, the King of Minyue, built it as an imperial city. The ruins of the Imperial City of the Minyue Kingdom, the “Best-Preserved Imperial City of the Western Han Dynasty (B.C. 206–A.D. 23) in the Pacific Rim Countries”, have a history spanning 2200 years.

**Figure 2:**
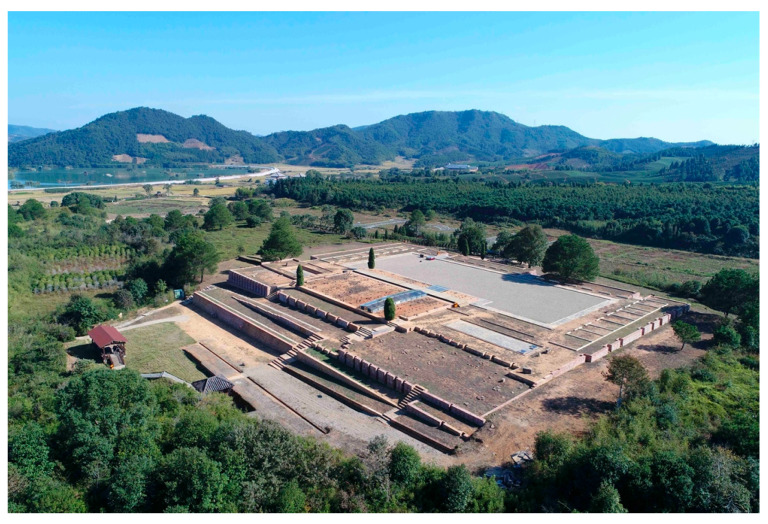
A panoramic photo of the ruins of the Imperial City of the Minyue Kingdom (image source: the 44th World Heritage Conference).

The Minyue Kingdom City is a relatively well-preserved ancient city site of the Han Dynasty south of the Yangtze River in China. The city wall of this site is a rammed earth building with a circumference of 2896 m and two city gates. Discoveries in the city include four large-scale building complex foundations, five iron smelting workshop sites, fifteen residential areas, beacon towers, drainage systems, and ancient roads. The central building area is a group of large palace buildings, including a gate, guard house, main hall, west wing room, west hall, and east warm room. There are also scientifically scattered water facilities, which are large in scale and reflect the architectural achievements of the Minyue people in the Han Dynasty. It is unique in site selection, architectural techniques, and style. It is a representative and model of the capitals of local vassal states at that time, and it shows the style and features of the ancient civilization of the Minyue Kingdom (Figure 2). It is the most well-preserved, largest, and most important archeological site in southern China. The Fujian Provincial People’s Committee announced it as the first batch of cultural relic protection units in 1961, and the State Council announced it as the fourth batch of national key cultural relic protection units in November 1996. UNESCO listed it as a cultural site in Wuyi Mountain on the World Heritage List in December 1999.

### 2.2. Sample Selection

**Figure 3:**
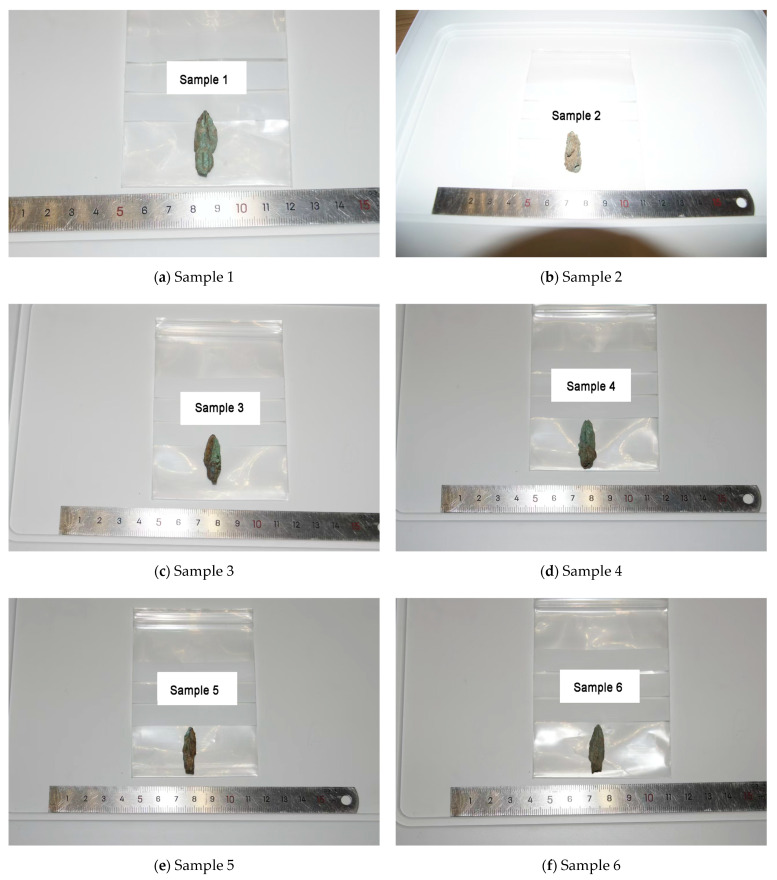

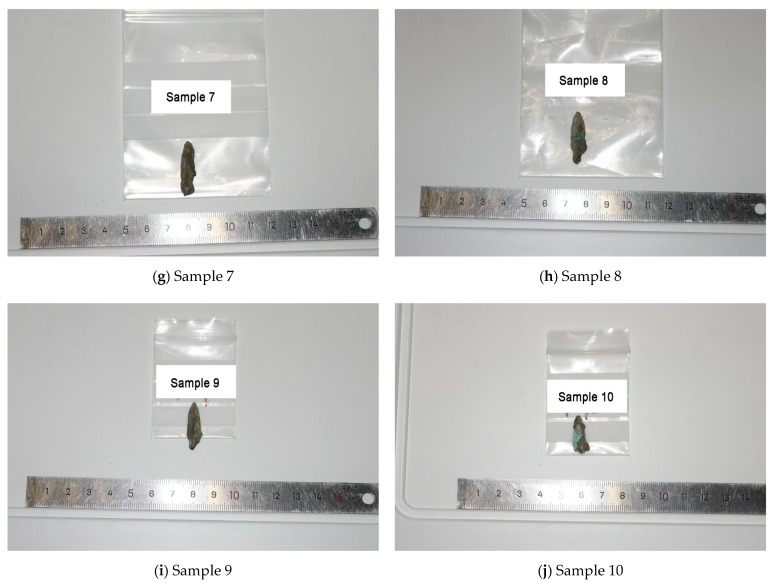
Researchers originally collected photos of 10 samples. The label on the packaging bag is the corresponding sample number (image source: photographed by the authors from the School of Civil Engineering and Architecture, Wuyi University).

When studying the bronze arrowheads unearthed from the ruins of the Imperial City of the Minyue Kingdom, the researchers divided them into three categories based on their different excavation sites. The gate guardhouse area yielded Type I arrowheads. These arrowheads may have been used as weapons by soldiers guarding the gates. Their quantity, shape, and preservation status are of enormous significance for studying the military defense system at that time. Type II arrowheads were unearthed in the foundry workshop area. These arrowheads may be waste or samples in the production process, providing us with valuable clues about the manufacturing process of ancient bronze arrowheads. As for Type III arrowheads, their excavation sites are scattered throughout the site. This study did not analyze Type III arrowheads due to their scattered use and generally poor preservation status. To further explore the characteristics of Type I and Type II arrowheads, the researchers selected samples from four city gates and four foundry workshops as typical for analysis in order to obtain more accurate and comprehensive research results (Figure 3).

It is worth noting that among the bronze arrowhead samples obtained from the ruins of the Imperial City of the Minyue Kingdom, their unearthed forms show significant differences. Specifically, these samples either appeared individually in a scattered form or in clusters or bundles. Through careful inspection of the unearthed locations, the researchers found that the samples located in the city gate guardhouse were mostly distributed in clusters, which may mean that these arrowheads were hoarded or stored here in some form before they were discovered. The soldiers manning the city gate had constant access to it. On the other hand, the scattered distribution characteristics of the samples found in the foundry suggest that they were either waste from the production process or abandoned within the workshop. This discovery offers fresh perspectives on the storage, use, and disposal of ancient bronze arrowheads.

**Figure 4:**
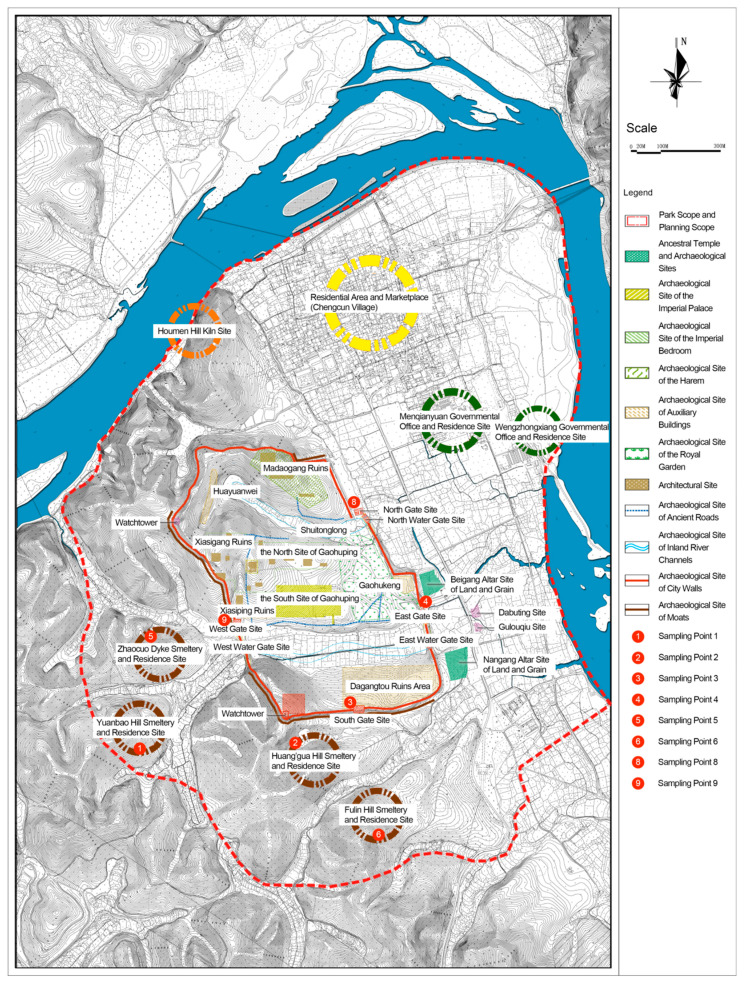
The distribution of locations where the 8 samples were unearthed is presented (image source: drawn by the authors).

Because samples 7 and 10 were damaged during the screening and handling stages, they could not be used for further studies during sample processing. In view of this, the researchers screened the samples based on their preservation conditions and finally selected eight samples in excellent condition and without obvious scratches for follow-up observation and analysis. This step ensured the accuracy of the study and the validity of the sample. Therefore, Figure 4 and Table 1 display the excavation points, excavation status, and selected cross-section directions of these eight samples.

### 2.3. Sample Processing

**Figure 5:**
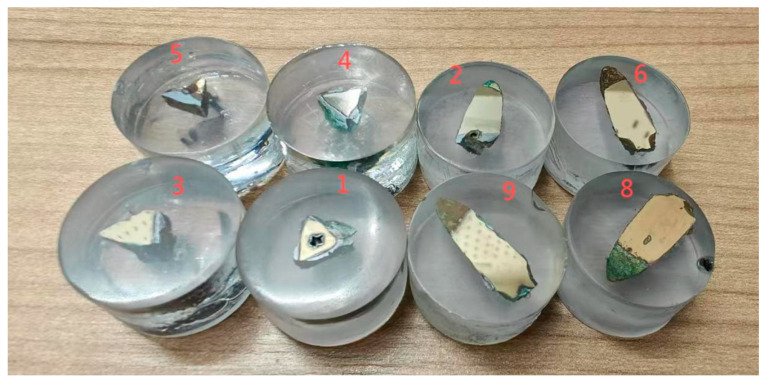
Photos of 8 samples after metallographic grinding (image source: photographed by the authors from the School of Civil Engineering and Architecture, Wuyi University).

The sample processing stage uses a systematic cleaning strategy to ensure the purity of the sample surface and the accuracy of the test (Figure 5). Within the rigorous scientific research framework, we followed the principle of fine processing from the outside to the inside and started pre-processing the selected samples. First, we used high-precision surface analysis tools, such as scanning electron microscopes (SEMs) and energy-dispersive spectrometers (EDSs), to obtain a full picture of the sample surface. This helped us to identify the different kinds of impurities and contaminants that were easy to work with on the surface. After that, we used micron-level precision manual operation tools to carefully remove these impurities and contaminants one by one. We made sure that every step of the process was performed with micron-level precision.

After removing any impurities on the surface, the researchers used a high-precision electrochemical workstation to precisely measure the oxide layer on the sample’s surface and determine its thickness and composition. Subsequently, nano-level polishing tools and precise mechanical control systems were used to manually and carefully peel off the oxide layer on the surface of the sample. A high-precision in situ ellipsometer was used to observe this process in real time to ensure that the oxide layer was removed with nano-level accuracy. This prevented any future precision test data from being obscured by oxide layers that were too thick on the surface. The dust and contaminants left after the initial cleaning may have comprised a variety of components, including inorganic matter, organic matter, chloride ions and their compounds, and other contaminants. These include the following: (1) inorganic matter: (a) soil particles: If the bronze arrowhead was buried in the soil for a long time before being unearthed, then soil particles may have adhered to its surface. (b) Metal oxides: during the long-term storage of the bronze arrowhead, it may have reacted with oxygen in the air to form metal oxides, such as copper oxide. (2) Organic matter: (a) microbial residues: microorganisms in the soil, such as bacteria and fungi, and their metabolites may have adhered to the surface of the bronze arrowhead. (b) Grease and dirt: the initial cleaning process may not have completely removed organic matter, such as grease and dirt. (3) Chloride ions: these are one of the harmful substances in bronze artifacts and may come from pollutants in the soil, groundwater, or air. Chloride ions may combine with bronze materials to form harmful substances, such as Cu_2_(OH)_3_Cl (dicopper chloride trihydroxide).

After the first cleaning step, the researchers used anhydrous ethanol as the solvent for ultrasonic cleaning. The strong vibrations of the ultrasonic cleaning machine removed all the dust and pollutants that remained on the sample’s surface. In order to verify the cleaning effect, high-precision surface analysis instruments were used to characterize the cleaned samples again. By comparing the data before and after cleaning, we confirmed that the cleanliness of the sample surface met the expected standards.

Secondly, the cleaned samples were fixed and the reserved position on the substrate was precisely cut. The researchers immediately dripped curing glue after cutting to ensure the samples’ stability and integrity. An automatic grinding and polishing machine (QATM Saphir560, Mammelzen, Germany) was then used to finely polish the samples. During the polishing process, we sequentially used sandpapers with mesh sizes of 100, 800, 2000, and 4000, along with polishing fluids ranging from 2.5 microns to 0.5 microns, operating at a pressure of 15 N and a rotation speed of 400, until the samples were fully polished. The researchers used diluted anhydrous ethanol as the polishing fluid and constantly checked the surface state of the samples during polishing to ensure that the polishing effect reached the mirror level. The samples were carefully inspected under an optical microscope (Olympus BX51M, Tokyo, Japan) with an accuracy of 50–100–200 μm to ensure that no obvious scratches were present.

Lastly, a metallographic vibratory polisher was used to apply the final treatment to the samples. This method uses vibrations to make tiny changes on the sample surface. It then uses the connection between the frequency of the vibration and the friction force on the object surface to apply vibrational force to the sample’s surface, which removes surface flaws, such as burrs, oxides, and scratches. The sample’s surface becomes smoother and more delicate after this processing step, and its surface morphology and structure also significantly improve, greatly facilitating subsequent observation and analysis work.

## 3. Results

### 3.1. Metallographic Analysis

#### 3.1.1. Imaging Analysis with a Metallographic Microscope

The researchers strictly followed the standard processing procedures and placed the eight polished slice samples of bronze arrowheads under a high-performance metallographic microscope (Olympus BX51M-Japan) for detailed microstructural observation. The magnifications were 50, 100, 200, and 500 times, respectively (Table 2).

At a low magnification of 50 times, the researchers first obtained an overall overview of the sample, aiming to preliminarily identify its general structural layout and possible macroscopic defects. As the magnification was gradually increased to 100 and 200 times, the overall state of the inclusions could be observed more clearly, and this information was crucial for understanding the microstructure of the material and its relationship with performance. This analysis primarily concentrated on the morphology and characteristics of the metal inclusions within the sample. On this basis, we further increased the magnification of the microscope to a high-magnification observation mode of 500 times and observed and recorded the morphological characteristics of the defects, the spatial distribution law, and the specific types and precise positioning of the inclusions in a more refined perspective. This information holds great significance for gaining a deep understanding of the material’s microscopic mechanism, evaluating its long-term preservation state, and understanding its behavior in historical use, particularly when considering the material’s integrity, durability, and potential failure modes. The black spots in Table 2 represent inclusions inside the bronze material. The image also displays scatters. Compared with the spots, they are smaller and irregular in shape. These spatters could be either tiny particles that fall off for some reason or metal debris generated during polishing or cutting.

#### 3.1.2. Burial Environment and Corrosion Mechanism

When the researchers looked at the polished sections of eight bronze arrowhead samples, they could immediately observe that the sample body had less corrosion in the longitudinal section than in the cross-section. This suggests that the corrosion intensity of bronze arrowheads is affected by a variety of internal and external factors, which can be summarized into two categories: internal and external factors. (1) The internal factors mainly relate to the inherent properties of the bronze arrowheads themselves. These include their material properties, such as alloy composition and microstructure; the influence of the manufacturing process, such as the casting technology or heat treatment process; and the state before burial, such as whether it had been surface-treated or whether there were initial defects or internal stresses. These factors together determine the ability of the arrowhead to resist corrosion during long-term burial. (2) The external factors include a variety of chemical and physical conditions directly related to the burial environment. The chemical factors include the soil pH, salt content, and redox reactions that are triggered. These conditions directly affect the chemical reaction rate on the surface of bronze arrowheads and the formation of corrosion products (such as CuO, ${Cu}_{2}O$, and $Cu(OH{)}_{2}CO_{3}$). The physical factors include the soil moisture, temperature, particle size and distribution, and the pressure exerted by the soil on the arrowheads. As the bronze arrowheads were unearthed from Wuyishan City, Fujian Province, China, the archaeological site is located in the mid-subtropical marine monsoon climate zone, which has four distinct seasons, abundant rainfall, no extreme heat in summer, and no severe cold in winter. Generally speaking, the fluctuations in soil temperature are smaller than those in air temperature, and due to the heat preservation effect of soil, the lowest soil temperature is often higher than the lowest air temperature in the same time period, and the highest soil temperature is often lower than the highest air temperature in the same time period. According to historical meteorological records, the average temperature in Xingtian Town, where the unearthed site is located, is roughly between 6 °C and 17 °C in winter, with extreme low temperatures of −4 °C to −2 °C; the average temperature in summer is usually around 25 °C–30 °C, and the extreme high temperature is typically 32 °C–33 °C. Combining the existing temperature records, the characteristics of the mid-subtropical marine monsoon climate zone, the vegetation coverage rate of the ruins of the Imperial City of the Minyue Kingdom, and other conditions, it is inferred that the soil temperature fluctuation range of the ruins of the Imperial City of the Minyue Kingdom is generally 5 °C~30 °C. These factors indirectly impact the corrosion process by influencing the diffusion rate of the corrosive medium and the shedding process of the corrosion products. Furthermore, we cannot ignore the influence of microbial activity and the alternation of groundwater and surface water as external factors. They further affect the corrosion behavior of bronze arrowheads by promoting or inhibiting specific biochemical reactions.

The stresses on bronze arrowheads in a buried environment are primarily caused by physical factors, such as soil pressure and temperature fluctuations. The different directions of the arrowhead unevenly distribute these stresses, leading to the corrosion behavior varying across different cross-sections. To be more specific, the arrowhead’s main force direction is along its length. This means that, during the casting process, its internal structure may have formed a denser and more uniform tissue arrangement, which improved its mechanical properties and resistance to corrosion in this direction. Therefore, compared with those on the cross-section, cracks on the longitudinal section do not form as easily, and the degree of corrosion is relatively low.

The mechanism of bronze corrosion essentially involves the process of smelting copper ore in its natural state with other metallic elements to form alloy copper. This alloy was subsequently cast into various artifacts. Under the combined effects of complex and changeable soil and geological conditions, the alloy gradually underwent the above-mentioned series of complex physical and chemical changes, resulting in the appearance of bronze artifacts after a long underground period. The interior gradually returns to a state akin to that of the original copper ore. Significant copper deterioration and the appearance of mineralization accompany this process. Under certain environmental conditions, the rust formed on the surface of the bronze will mineralize over time, forming unique crystallization traces. These mineralized crystals not only record the long history of the interaction between bronzes and the environment but also form an important scientific basis for identifying the age of bronzes, evaluating their preservation status, and studying corrosion mechanisms in the fields of archeology and cultural relic identification.

#### 3.1.3. Metallographic Structure Characteristics

The researchers also looked closely at the metallographic structural features of the eight samples and made a list of important details, such as the type, number, size, and location of any possible flaws or inclusions (Table 3).

Through comparative analysis, we can find that the size and shape characteristics of the inclusions in these eight samples are similar. These samples commonly contain inclusions of various sizes, primarily taking the form of point-shaped and irregular inclusions, with a relatively uniform distribution on the cross-section. Subsequent analysis revealed that the following three factors could potentially influence the variation in inclusion size.

First of all, the smelting raw materials and processes have an important influence on the formation of inclusions. Ancient bronze smelting mainly used natural ores as raw materials. In addition to being rich in copper, these ores also contain a variety of impurity elements, such as sulfur, iron, and lead. The smelting process may not completely remove these impurity elements, leading to the formation of inclusions. In addition, conditions, such as temperature control and redox reactions during the smelting process, also affect the formation of inclusions. For instance, an insufficient smelting temperature or redox reaction may result in incomplete separation of the impurity elements from the copper matrix, leading to the formation of inclusions. In general, the inclusions in this complex process include the following: (1) metal elements and their compounds: (a) copper (Cu): as the main component of bronze, copper may exist in inclusions in the form of pure copper or copper alloy. (b) Tin (Sn): an alloying element often added to bronze, it may also exist in inclusions and form copper–tin alloys with copper. (c) Lead (Pb): some bronze products may contain lead, and lead and its compounds may appear as inclusions. (d) Other metal elements, such as zinc (Zn) and iron (Fe). These elements may exist in bronze arrowheads in trace amounts and may form inclusions. (2) Non-metallic elements and their compounds: (a) oxygen (O): combines with metal elements to form oxides, such as copper oxide (CuO, Cu_2_O, etc.). (b) Sulfur (S): it may exist in the form of sulfides, such as cuprous sulfide (Cu_2_S). Sulfide inclusions are common in bronze artifacts and may affect their performance. (c) Arsenic (As): some bronze artifacts, especially those from specific regions, such as those unearthed in Chifeng, western Liaoning, China, may contain arsenic. Arsenic and its compounds may appear as inclusions. (d) Silicon (Si), phosphorus (P), etc.: These elements may exist in inclusions in the form of silicates, phosphates, etc. (3) Other compounds: (a) chlorides: The reaction of chloride ions in the soil with copper in bronze artifacts can form such compounds as copper chloride (CuCl). (b) Carbonates, such as lead carbonate (PbCO_3_), may reveal the lead corrosion process.

Secondly, the casting process of bronze arrowheads is also a key factor affecting the distribution and shape of inclusions. Various factors, such as the temperature gradient, metal fluidity, and shape and material of the mold affect the molten metal as it cools and solidifies in the mold during the casting process, potentially causing changes in the distribution and shape of inclusions in the metal matrix. In particular, the speed of cooling significantly impacts the distribution and morphology of inclusions. A faster cooling rate may prevent inclusions from diffusing and aggregating, resulting in the formation of smaller and evenly distributed inclusions, while a slower cooling rate may cause inclusions to aggregate and grow.

Finally, we cannot ignore the environmental corrosion effect of bronze arrowheads during the burial process. Environmental factors, such as soil, moisture, and oxygen, corrode the arrowheads, leading to oxidation on the surface of the inclusions, the formation of corrosion products, and interfacial reactions between the inclusions and the matrix. These corrosion effects can affect the morphology and distribution of inclusions. At the same time, different storage environments also impact the corrosion degree and speed of bronze arrowheads. For example, arrowheads buried in moist, acidic, or alkaline soils may corrode at a higher rate and to a greater extent, resulting in more significant changes in the morphology and distribution of inclusions.

In addition, it is worth noting that in the four samples of the longitudinal section, the distribution of inclusions is relatively uniform, while in the cross-sectional samples No. 6 and No. 8, the distribution of inclusions shows obvious stratification. This may also be related to the above three reasons. Additionally, the later stage of the arrowhead’s sharpening process may have accelerated the arrowhead’s rust process.

### 3.2. SEM–EDS Analysis

**Figure 6:**
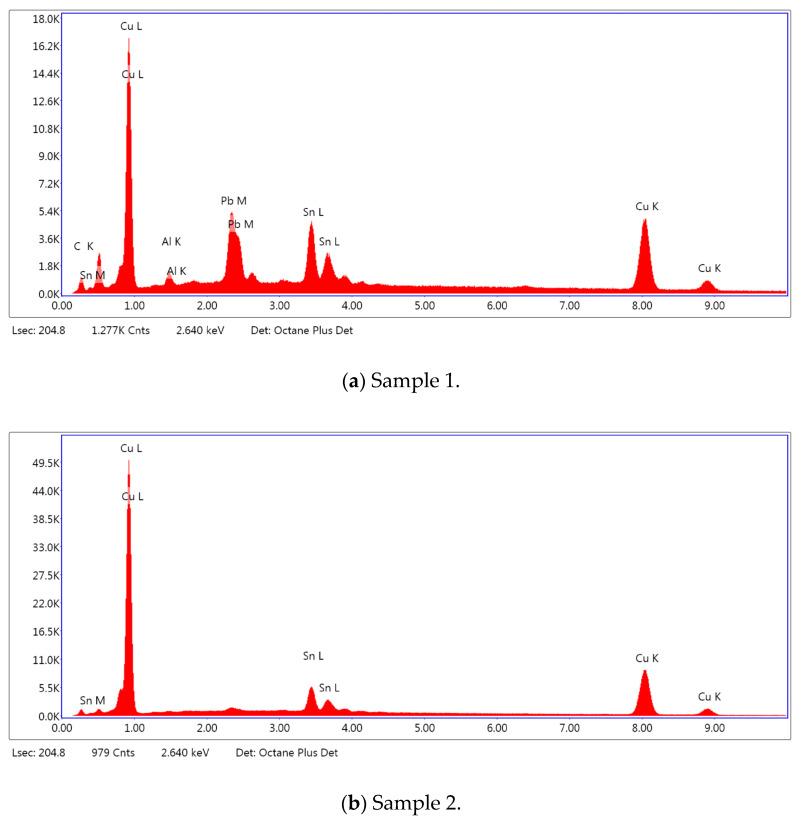

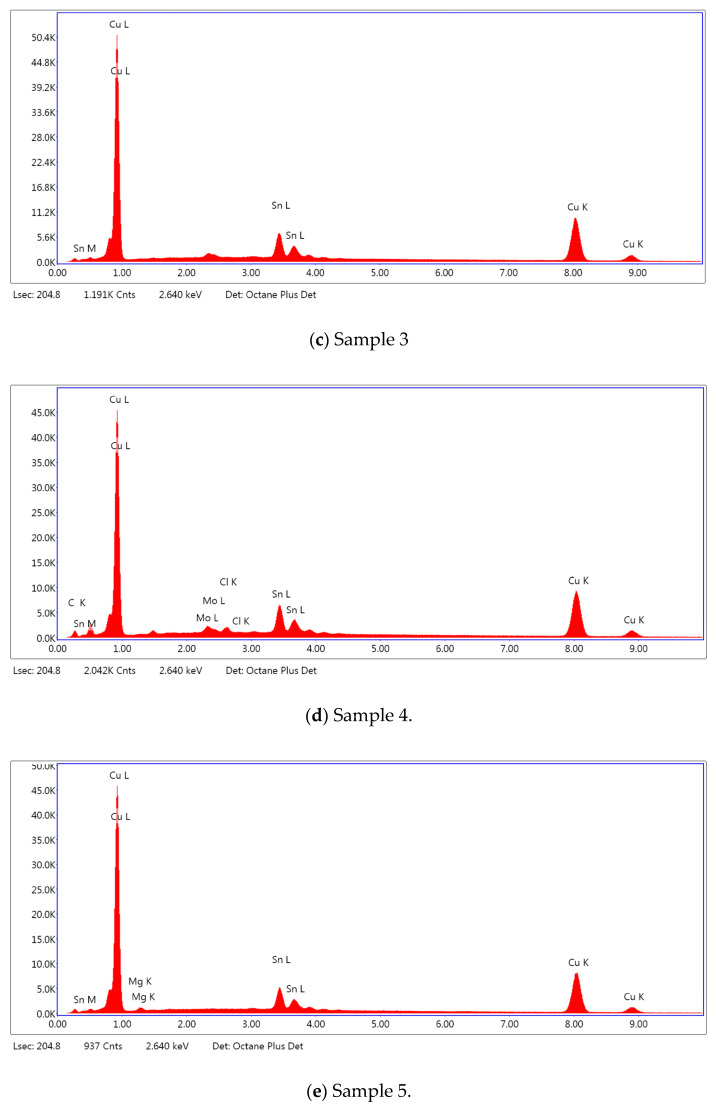

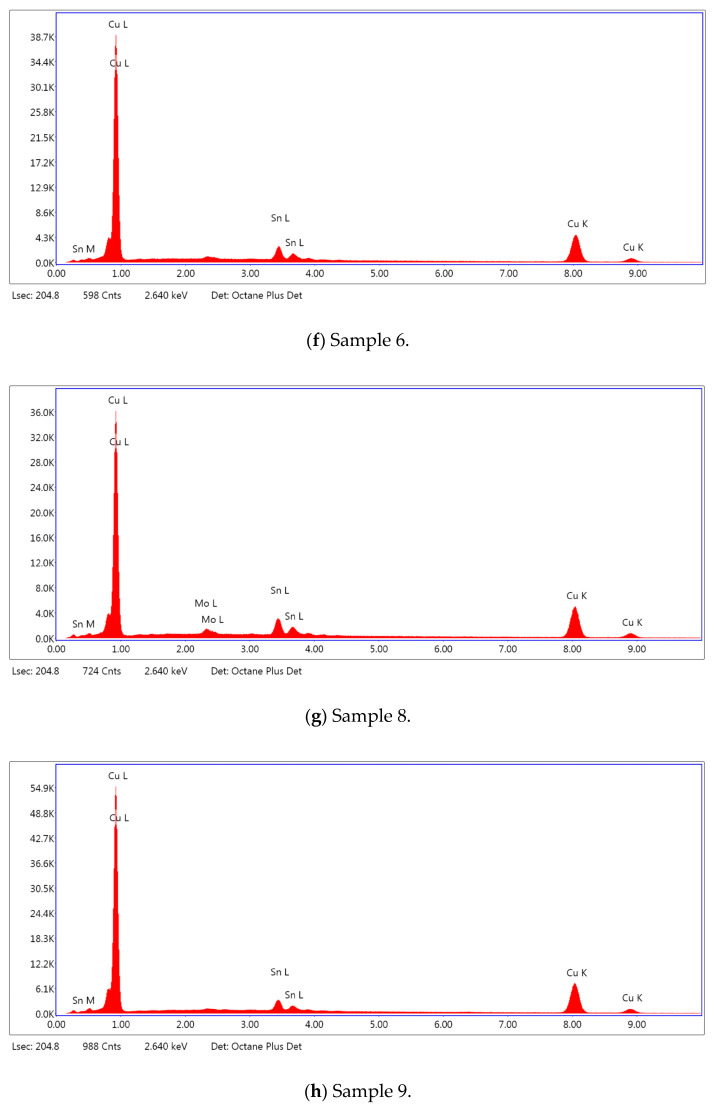
Element distribution of 8 bronze arrowhead samples (image source: compiled by the author based on energy-dispersive spectrometer analysis results).

Following metallographic analysis, the researcher sliced and polished the eight samples again, observed them using a scanning electron microscope (SU8000), and determined the sample composition using an energy-dispersive spectrometer. The primary objective was to examine the parts that had a stable composition and state. The electron acceleration voltage of the scanning electron microscope was 20.0 keV, the working distance was 10.4 mm, and the magnification was 5039 times. Table 4 and Table 5, and Figure 6 display the element distribution of the eight samples, while Table 6 displays the results of the composition analysis. Table 7 shows the eZAF smart quant results.

#### 3.2.1. Element Distribution Analysis

Table 4 shows the scanning electron microscope (SEM) images of eight bronze arrowhead samples and their element distribution overlays, revealing significant differences in the microstructure and element distribution among different samples. The surface of sample 1 presents irregular large granular features, and lead (Pb) is concentrated in the element overlay diagram (the red area in the figure represents lead), which may be due to deliberate addition during the casting process to improve the fluidity of the alloy. Samples 2 and 3 have relatively flat surfaces and evenly distributed pores, and they are mainly composed of copper (Cu) and tin (Sn) (the cyan area in the figure represents copper, and the yellow area represents tin), indicating that they are copper–tin binary materials. In the alloy, no lead element was found. The surface of sample 4 showed stripe corrosion and cracks. The grains of samples 5 and 6 were fine and uniform, showing the casting characteristics of rapid cooling, while obvious molybdenum (Mo) was detected in the crack area of sample 8 (the blue area in the figure represents Mo), which may be related to the special burial environment or may depend on the soil conditions. Sample 9, like the other samples, is a copper–tin binary alloy with a homogeneous structure, indicating a consistent casting process.

Table 5 shows the element distribution characteristics of the eight bronze arrowhead samples in detail. Energy-dispersive spectrometry (EDS) shows the distribution of elements, such as C, Al, Pb, Sn, Cu, Mo, Cl, and Mg, on the sample surface. In sample 1, the lead (Pb) distribution is the most significant, and together with tin (Sn) and copper (Cu), it forms a more obvious regional aggregation, indicating that a higher proportion of lead may be added during the casting process. The alloy fluidity and casting properties could be improved. The appearance of carbon (C) may be related to the organic matter residue in the burial environment. Tin (Sn) and copper (Cu) are evenly distributed in all the samples, and no obvious lead (Pb) content is detected in the samples except sample 1, which further confirms that these samples are typical copper–tin binary alloys. Based on the distribution of copper (Cu) and tin (Sn), samples 4 and 8 were found to have small amounts of molybdenum (Mo) and chlorine (Cl), respectively, which may be closely related to the soil environmental characteristics and human activities during their burial. In particular, molybdenum (Mo) may be affected by agriculture through the impact of fertilizers or industrial pollution.

#### 3.2.2. Energy-Dispersive Spectroscopy (EDS) Analysis

Figure 6 shows the energy-dispersive spectroscopy (EDS) analysis results for the eight bronze arrowhead samples, illustrating the presence and distribution characteristics of the main elements in each sample one by one. In the EDS spectrum of sample 1, copper (Cu) and tin (Sn) are the main components, and show significant distribution characteristics at the peak. In addition, the peak value of lead (Pb) is relatively high, indicating that the sample contains a significant proportion of lead, which may be related to the deliberate addition of lead during the casting process to improve the fluidity of the alloy and lower the melting point. The trace amounts of carbon (C) and aluminum (Al) may have originated from the burial environment or impurities during casting. Small amounts of molybdenum and chlorine were detected in samples 4 and 8, which may be related to the complexity of the burial environment. Copper (Cu) and tin (Sn) account for the main proportions of all the samples and are evenly distributed, indicating that these samples are relatively pure copper–tin binary alloys. The composition characteristics further support the hypothesis that they may come from the same production process or batch.

Table 6 shows the main chemical compositions and mass fractions (wt%) of the eight bronze arrow samples based on energy-dispersive spectroscopy (EDS) and details the chemical composition of each sample in terms of copper (Cu), tin (Sn), lead (Pb), carbon (C), and other trace elements, providing support for a deeper understanding of its casting process and burial environment. (1) In sample 1, the copper (Cu) content was 68.75%, the tin (Sn) content was 15.54%, the lead (Pb) content reached 11.19%, the carbon (C) content was 4.08%, and a trace amount of aluminum (Al 0.45%) was detected. This sample had a significantly higher lead content than the other samples, suggesting that lead may have been added during the casting process to improve the fluidity or lower the melting point. The high carbon content may be related to organic matter residues in the burial environment. (2) In sample 2, copper (Cu) accounted for 87.79%, tin (Sn) accounted for 12.21%, and no lead (Pb) or other impurities were detected. Its composition is relatively pure, and it is a typical copper–tin binary alloy, indicating that its casting process is stable and has not been significantly polluted by the environment. (3) In sample 3, copper (Cu) was 87.02% and tin (Sn) was 12.98%, while no lead (Pb) or other impurities were detected. The composition was highly consistent with that of sample 2, indicating that the two may have come from the same production batch or casting process. (4) In sample 4, copper (Cu) was 82.77%, tin (Sn) was 12.87%, and carbon (C) was 3.04%. A small amount of molybdenum (Mo 0.79%) and chlorine (Cl 0.53%) were detected. The presence of molybdenum and chlorine may be related to the complex chemical environment of the buried soil or human activities (the fertilizers used in agriculture) and may reflect specific corrosion behavior. (5) In sample 5, copper (Cu) was 86.96%, tin (Sn) was 12.12%, and a small amount of magnesium (Mg 0.92%) was detected. The magnesium may have come from soil minerals or groundwater infiltration in the burial environment, indicating that environmental influences influence its corrosion characteristics. (6) In sample 6, copper (Cu) was 88.79% and tin (Sn) was 11.21%; there were no other significant impurities. The purity of the composition indicates that its casting process was stable, which is conducive to the production of high-quality weapons. (7) In sample 8, copper (Cu) was 86.00%, tin (Sn) was 12.50%, and a small amount of molybdenum (Mo 1.50%) was detected. The high content of molybdenum may indicate that its burial environment was affected by specific soil chemical characteristics, such as agricultural fertilizers or industrial pollution. (8) In sample 9, copper (Cu) was 91.34%, tin (Sn) was 8.66%, and there were no other significant impurities. The higher copper content may indicate that the casting process of this sample focused on improving the alloy toughness to meet specific usage requirements.

#### 3.2.3. The eZAF Smart Quantitative Analysis

Table 7 provides the eZAF smart quantitative analysis results for the eight bronze arrow samples, including the mass percentages (Weight %), atomic percentages (Atomic %), and measurement errors (Error %) of the main elements. The measurement error (Error %) is a relative error range estimated based on comprehensive factors, such as the EDS signal intensity (the weaker the signal, the higher the error), background noise, instrument performance, and algorithm correction. The following can be observed from Table 7: (1) the copper atomic percentage of sample 1 (66.67%) was significantly lower than that of sample 2 (93.07%) and sample 3 (92.60%), which indicates that the alloy design of sample 1 was more inclined to lead (Pb) and tin (Sn) to optimize the casting properties, while samples 2 and 3 prioritized high copper ratios to improve toughness and strength. (2) The molybdenum (Mo) content of sample 4 was 0.79%, and the chlorine (Cl) content was 0.53%, while the molybdenum (Mo) content of sample 8 was 1.50%, indicating that the molybdenum (Mo) and chlorine (Cl) contents may have come from chemical migration during long-term burial, especially the significant increase in the molybdenum (Mo) content in sample 8, suggesting that the burial environment of the sample was subject to stronger external chemical disturbances. (3) The high error values (e.g., 16.75% for aluminum (Al) and 10.78% for carbon (C) in sample 1) suggest that these impurities may have been the result of random infiltration from the environment. The main components with lower errors (e.g., the errors for copper and tin are generally in the range of 3–4%) show the high consistency and stability of the main alloy composition of the samples. (4) The atomic percentages of carbon (C) in samples 1 and 4 were 20.91% and 15.00%, respectively, and the mass percentages were 4.08% and 3.04%, respectively, which were significantly higher than those of the other samples. The precise quantification of these data suggests the potential impact of organic matter in the burial environment on the surface of the material, while the lack of significant carbon content in other samples suggests that the surfaces of these samples have been relatively well-preserved and have not been extensively eroded by organic matter. (5) The atomic percentage of magnesium in sample 5 was 2.50%, with a high deviation (13.49%). This indicates that the source of magnesium (Mg) was somewhat random and may have only existed on the surface of the sample. Its formation mechanism is more likely to be related to the short-term migration of soil minerals or water-soluble salts.

#### 3.2.4. Comprehensive Analysis and Inference Results

Through electron microscope observation, researchers found that the internal texture of Han Dynasty bronze arrowheads showed a complex organizational structure. First, on the polished section of the arrowhead, a rust layer composed of morphological mineral layers can be clearly observed. These mineral layers intertwine to form a unique rust structure. Further observation revealed that the internal tissue structure of the arrowhead consists of three parts: a completely mineralized layer, an intermediate transition layer, and the original heart tissue. The outermost layer contains the completely mineralized layer, which is primarily composed of corrosion products, like patina and copper oxide. Its thickness and shape show significant diversity due to differences in corrosion degree and time. The intermediate transition layer exhibits more complex structural characteristics, including mineral particles of different shapes (such as needle-shaped, flaky, and spherical) and pores (such as micropores and cracks). Phenomena, such as material migration, rearrangement, and phase changes during corrosion, may closely relate to the formation of these features. The heart’s original structure preserves the microstructural features of the cast arrowhead, including the grain size, shape, and distribution. The observation process also revealed potential defects and abnormal structures inside the arrowhead. These defects include inclusions (such as non-metallic inclusions and intermetallic compounds), pores (such as casting pores and corrosion pores), and cracks (such as hot cracks and cold cracks). Various factors, such as improper workmanship during the casting process, material quality issues, or damage during later use, may have caused these defects to exist.

Furthermore, the results of the SEM observation and composition analysis fundamentally suggest that samples 2, 3, 4, 5, 6, 8, and 9 are copper–tin binary bronze arrowheads. Sample 1 tested positive for Pb, with a proportion as high as 11.19%. The researchers proposed two possible explanations. First, lead may have been added to the arrowhead during the casting process as an alloy or other material, making it part of the arrowhead before it was fully cast. Second, because the East Gate of the ruins of the Imperial City of the Minyue Kingdom may have had more severe environmental pollution, the researchers thought that, after the sample had been used, the lead element may have gotten into the bronze arrowhead through element replacement between the soil and the bronzeware. Both hypotheses require more in-depth investigation and experimental verification in the future to reveal the real reason for the high lead content in sample 1.

During a detailed composition analysis of bronze arrowhead samples unearthed from the ruins of the Imperial City of the Minyue Kingdom, researchers found that the results for samples 2, 3, and 6 were highly consistent. Only two elements, namely copper (Cu) and tin (Sn), were detectable within the monitoring accuracy of the instrument, with the mass ratios of the copper elements in each sample being 87.79%, 87.02%, and 88.79%, respectively. These data show that the three samples have very similar composition ratios and structures, suggesting that they may have come from the same batch of production or supply. The researchers speculated, based on this discovery, that the arrowheads used in the Huang’gua Hill Smeltery and Residence Site, the Fulin Hill Smeltery and Residence Site, and the South Gate might have originated from the same batch, offering new insights into the production and distribution of ancient Chinese weapons.

However, upon comparing other samples, the researchers detected the element molybdenum (Mo) in both samples 4 and 8. Considering that the east side of the ruins of the Imperial City of the Minyue Kingdom is close to residential areas, this geographical feature may have led to complex changes in the soil environment. Human activities, such as the use of chemical fertilizers in agricultural farming, industrial emissions, and natural factors, may have a significant impact on the physical and chemical properties of soil, such as its pH and redox status, thereby interfering with the natural cycle and distribution of elements in the soil. Therefore, we speculate that the unique soil environment and human activities in this area may closely relate to the occurrence of molybdenum in samples 4 and 8.

There are three main possible reasons for the presence of small amounts of C, Al, Mo, Mg, and Cl in the samples. (1) One possible explanation is that the bronze arrowheads may have corroded during burial, leading to the generation of various corrosion products. These corrosion products may have contained elements from the original alloy or from the environment. Certain elements, such as Mo and Mg, may have come from impurities or added trace elements in the alloy. During the corrosion process, these elements may have existed or emerged as specific compounds. Cu reacts with Cl to generate CuCl, which is a common product in the corrosion process of bronze. (2) It may be that the bronze arrowheads were buried underground for a long time, and elements in the soil, such as C (possibly from organic matter in the soil) and Cl (possibly from salt in the soil or groundwater), entered the interior of the arrowheads through infiltration, diffusion, etc. (3) During the excavation process, improper protection measures or unprofessional handling methods may have contaminated the bronze arrowheads, leading to the introduction of additional elements. For example, excavation tools, storage containers, or processing reagents may contain certain elements, such as C, Al, Mo, Mg, and Cl.

However, the test results did not reveal nickel (Ni). Previous studies have shown that nickel has not been found in ancient Chinese bronze arrowheads [12], although it is often a coexisting element in copper alloys. The reasons for this phenomenon may include the following: (1) smelting technology and raw materials: the smelting technology of bronzeware in the Han Dynasty was relatively limited, and it may not have been possible to accurately control the element content in the alloy. The raw materials for bronze arrowheads in the Han Dynasty may have mainly come from local copper ores, which may have had a low nickel content, which would have made it impossible to form sufficient nickel content during the smelting process. (2) Alloy formula and purpose: the production of bronze arrowheads in the Han Dynasty may have focused more on their hardness and toughness to meet actual combat needs. Although nickel (Ni) can enhance the hardness and corrosion resistance of bronze, it may not have been necessary for making arrowheads in the Han Dynasty. Han Dynasty craftsmen may have preferred to use other alloying elements (such as tin and lead) to adjust the properties of bronze. (3) Preservation environment and corrosion: Han Dynasty bronze arrowheads may have experienced a long period of burial and corrosion before being unearthed. Such corrosion may have caused changes in the element distribution on the surfaces of the bronze arrowheads and may have even covered up the nickel (Ni) element in the original alloy. The alloy may have still contained nickel after this loss but at a less detectable level. (4) Causes of deviation in detection methods and sensitivity: the detection method and sensitivity of electron microscopy analysis may present limitations in the detection of nickel. Electron microscopy analysis might not have allowed the detection of nickel in the bronze arrowhead if it was very small or in a form that was hard to see, such as when combined with other elements. Further research or comparative analysis with other comparable artifacts may provide a clearer explanation for this phenomenon.

## 4. Discussion

### 4.1. Interpretation of the Casting Process of Bronze Arrowheads

The metallographic and SEM energy spectrum tests on the eight samples reveal that the bronze arrowheads found in the ruins of the Imperial City of the Minyue Kingdom are mostly made of copper–tin binary alloys. The matrix is a typical α-dendritic crystal. The metallographic structure lacks equiaxed crystals or twins, suggesting that the bronzeware underwent casting.

The alloy composition of bronzes usually involves a copper–tin binary alloy system or a more complex copper–tin–lead ternary alloy system. In these alloys, changes in the contents of copper, tin, and lead elements have significant and complex effects on the physical and mechanical properties of the bronzes. Specifically, the balance of toughness and hardness in bronze directly correlates with the content of copper, which is the main component of bronze. Generally speaking, the higher the copper content, the more the toughness of the bronze increases, while the hardness decreases. To optimize the overall performance of bronzewares, it is often necessary to add tin, lead, and other alloying elements to copper.

The addition of tin is particularly critical to improving the performance of bronzeware. It can not only effectively lower the melting point of the alloy, making the casting process more cost-effective, but also significantly improve the strength and hardness of bronze. In particular, when the tin content is in the range of 5% to 10%, the hardness of bronze increases significantly, which is particularly important for manufacturing objects that need to withstand certain external forces or wear. However, when the tin content exceeds the 10% threshold, the toughness of the bronze decreases significantly and the brittleness increases. This results in reduced tensile strength and elongation, increasing the risk of fracture, which poses a threat to the long-term stability and service life of the bronze.

Lead plays a crucial role in bronze alloys. It can significantly improve the fluidity of the alloy, making it easier for bronzes to fill complex mold structures during the casting process and thereby improving yield and detail expression. Moreover, lead also contributes to lowering the melting point, albeit to a lesser extent than tin does. However, excessive lead content has a negative impact on the hardness and corrosion resistance of bronze, not only reducing its mechanical strength but also affecting its aesthetics and practicality. Therefore, the amount of lead in the alloy mix needs to be precisely controlled to prevent the alloy from becoming too brittle while maintaining fluidity. At the same time, an appropriate amount of lead can also be used as a substitute for tin to adjust the brittleness of the alloy under specific circumstances to meet the needs of different application scenarios.

According to the *Zhou Li·Kaogongji* records, the gold of bells and tripods is six parts, and tin is one part; the gold of axes and knives is five parts, and tin is one part; the gold of halberds and halberds is four parts, and tin is one part; the gold of large blades is three parts, and tin is one part; the gold of arrowheads is five parts, and tin is two parts; the gold of flints and arrows is half and half. This shows that the manufacturing technology of bronzeware in China during the Warring States period had reached a certain maturity. Regarding the discussion of the composition of arrowheads, there are many forms of expression in historical documents. Among them, there are two more common interpretations: one is that copper accounts for 40% and tin accounts for 60% of the total content. The modern mathematical concept directly converts this ratio, while the unique fractional expression from ancient times interprets the copper–tin ratio as three-fifths and two-fifths, respectively, with copper accounting for 60% and tin for 40%. In addition, there are records of approximate ratios, such as 7:3 and 6:4. These differences may be due to errors in the process of copying documents or different understandings and practices regarding alloy ratios in different regions and periods. Modern materials science research points out that a copper–tin alloy (i.e., bronze), in which copper accounts for 70–80% and tin accounts for 20–30%, can most effectively balance the hardness and toughness of the arrowhead, ensuring that it has sufficient penetrating power in actual combat and can withstand a certain amount of impact without breaking easily, thereby achieving the best attack effect.

**Figure 7:**
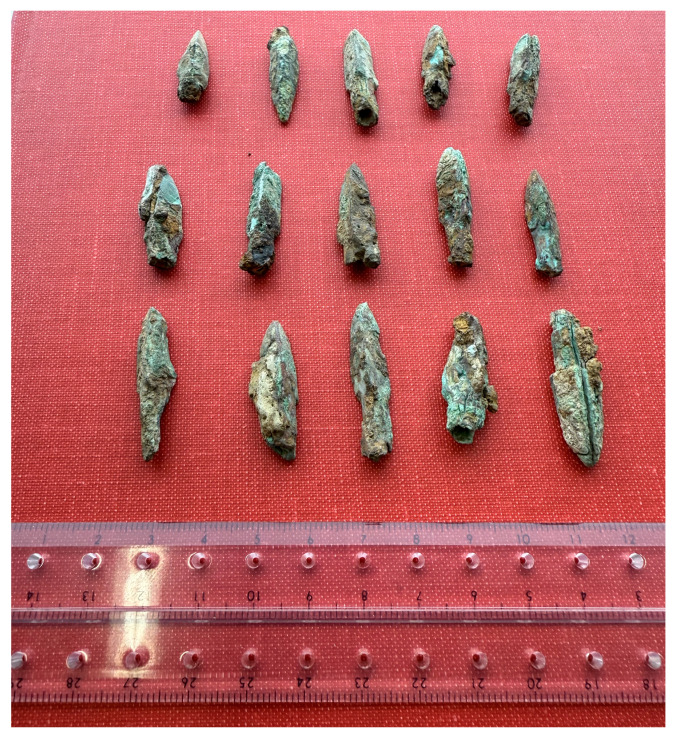
The shape of the bronze arrowhead (image source: photographed by the authors from the School of Civil Engineering and Architecture, Wuyi University).

**Figure 8:**
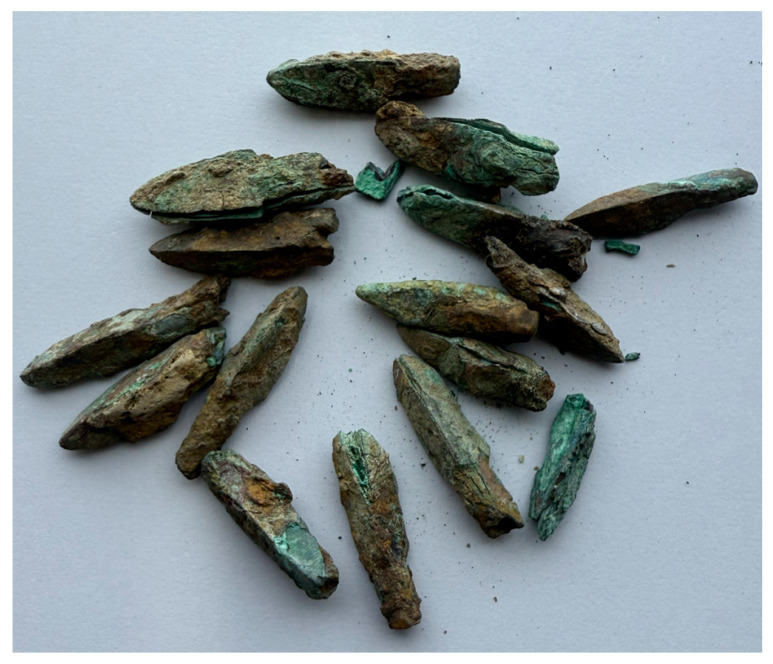
Bronze arrowheads unearthed in batches (image source: photographed by the authors from the School of Civil Engineering and Architecture, Wuyi University).

According to archeological excavations and research in recent years, more than 2000 years ago, China had already seen the emergence of industrial production line molds to produce standardized arrowheads (Figure 7), as well as a large number of typical three-edged arrowheads (Figure 8). This discovery not only shows that, in ancient times, the production of bronze arrowheads had achieved a high degree of standardization and scale but also reveals the advanced technology and organizational capabilities of ancient China in the field of weapon manufacturing. This also explains why arrowheads show a certain regularity and consistency in terms of internal organizational structure, grain morphology, and distribution, which further confirms the exquisite craftsmanship and strict quality control of ancient China in the manufacture of bronze weapons.

Therefore, the researchers further speculated that the Minyue Kingdom had accumulated some experience in bronze forging technology during the Han Dynasty, approximately 2000 years ago, particularly in the control of alloy composition. However, despite having mastered the processing technology of bronze materials to a certain extent, the Minyue Kingdom continued to explore and practice the application of weapons, particularly focusing on the alloy ratio of arrowheads, a crucial component. This shows that, even in ancient civilizations with relatively advanced technology, the optimization of alloy composition for specific purposes was a long and complex process.

In addition, the evaluation of arrowhead casting technology and military capabilities in different eras and countries requires the comprehensive consideration of multiple factors, including but not limited to the mining difficulty, the market prices of metal raw materials, technical exchanges, and differences between regions. These factors collectively influenced the selection of arrowhead alloy components and the evolution of manufacturing technology, allowing us to more comprehensively and objectively evaluate the development level of ancient weapon manufacturing technology and the socioeconomic background behind it. Therefore, in-depth research on ancient bronzes, especially weapons, such as arrowheads, not only helps to reveal the secrets of ancient science and technology history but also provides valuable clues for us to understand the economy, culture, and even military strategy of ancient society.

### 4.2. Measures to Protect Bronze Arrowheads from Metal Corrosion

Table 8 describes the restoration and protection measures that have been implemented and those that are planned for bronze artifacts for the eight bronze arrowhead samples taken as examples in this study. These measures comprehensively consider the material properties, surface corrosion conditions, and environmental sensitivity of the samples while focusing on achieving the long-term stability of the materials based on protecting the integrity of the artifacts. In the future, bronze artifacts may benefit from their application in similar situations.

(1) During the mechanical cleaning stage, the research team used two techniques: low-speed polishing and air abrasion. For sample 1, because the surface was found to have a dendritic structure rich in lead, a QATM Saphir 560 low-speed metallographic polisher and 4000-grit sandpaper were used for slow surface cleaning to avoid damaging the microstructure of the area. For samples 4 and 8, because their surface corrosion layers were relatively dense, low-pressure alumina powder air flow abrasion was selected to accurately remove soil particles and retain the corrosion product layer. This refined operation fully considered the surface conditions of different samples and demonstrated the focus on sample specificity in the restoration work.
(2) During the chemical cleaning stage, the research team adopted differentiated strategies for the corrosion conditions of different samples. The oxide layer of samples 6 and 9 was removed using a 5% EDTA solution combined with ultrasonic vibration. For the severely corroded local areas of sample 1, a 1% benzotriazole (BTA) solution was used to inhibit the further oxidation of copper–lead compounds. At the same time, all the samples were rinsed with deionized water with a neutral pH value after cleaning, which not only effectively neutralized the possible residual corrosive substances but also avoided the occurrence of secondary reactions. The method selected for chemical cleaning directly corresponded to the composition and corrosion product distribution characteristics detected in the EDS analysis of the samples, such as the high lead content of sample 1 and the slight oxidation phenomenon of sample 9.
(3) In terms of stabilization treatment, the research team adopted a variety of cutting-edge technologies to improve the chemical stability of the samples. For example, vacuum freeze-drying technology was used for samples 2 and 3 to completely remove adsorbed water at −50 °C, and ion-exchange resin was used to gradually reduce the content of CuCl_2_ to prevent the formation of malachite (Cu_2_(OH)_3_Cl). For samples 5 and 6, which had been cleaned, a layer of neutral sealant (Paraloid B-72) was applied to form an effective physical isolation layer, thereby inhibiting the intrusion of oxygen and moisture, and effectively preventing the propagation of microcracks in high-tin areas in particular. These measures directly target the chemical composition presented in Table 6 and Table 7 and the fragility of the sample microstructure, providing a scientific protective barrier for the samples.
(4) To ensure long-term preservation, the research team designed a strict environmental control plan for the samples. All the samples were stored in an environment with a relative humidity (RH) controlled at 40% to prevent Cu_2_Cl_2_ from further converting into malachite under high-humidity conditions. At the same time, sample 4 was stored in a low-oxygen environment and sealed in a nitrogen environment with an oxygen content of less than 5% to inhibit oxidation reactions, and a small amount of sulfur dioxide (SO_2_) was added to simulate the environmental stabilization process of molybdenum. These environmental control measures fully considered the chemical changes that the samples underwent during the burial process and, combined with experimental test data (such as the distribution of molybdenum and chlorine in samples 4 and 8), an optimized storage plan was developed.
(5) For high-corrosion risk areas (such as samples 1 and 8), the research team also introduced advanced corrosion inhibition technology. The method of local immersion in a 2% BTA solution further enhanced the stability of the passivation film on the sample surface, while the placement of volatile corrosion inhibitors (VCIs) in the storage environment of samples 5 and 6 effectively reduced the possibility of atmospheric corrosion. This multi-level protection method combining immersion and environmental regulation ensures the long-term stability of high-risk samples while retaining the original metallographic and surface properties to the maximum extent.

By comprehensively applying the above-mentioned multiple protection technologies and combining the composition characteristics and corrosion laws of the samples, effective protection of bronze arrowheads can be achieved, ensuring the long-term preservation of their academic research and cultural value.

## 5. Conclusions

### 5.1. Research Discoveries

This study conducted a systematic analysis of the bronze arrowhead samples unearthed from the ruins of the Imperial City of the Minyue Kingdom. This study conducted an in-depth analysis of four aspects—microstructure, chemical composition, casting process, and corrosion behavior—revealing the important material and historical value of these arrowheads. The main conclusions of this study are as follows:

(1) Characteristic analysis of microstructure: Based on high-resolution observations under a metallographic microscope, the researchers clearly observed that the main body of the copper arrowhead unearthed from the East City Gate warehouse showed typical copper–tin binary alloy characteristics, specifically the alpha-dendrite structure. The morphological parameters of this structure, such as the dendrite spacing and trunk length, were measured to be approximately 5 and 20 μm, respectively, directly indicating that the arrowhead was cast at a high temperature and did not undergo significant post-heat treatment. Combined with historical materials, the main process of bronze arrowhead casting in the Imperial City of the Minyue Kingdom was making a mud film, covering the mud mold, drying and burning the inner mold, melting bronze liquid, injecting the bronze liquid, cooling and removing the mold, polishing and finishing, and quality inspection. The step of ore smelting was usually completed before the casting process. Ore smelting is the process of refining copper ore into bronze materials that could be used for casting. It was an indispensable part of the bronze arrowhead casting process. During the ore smelting stage, craftsmen collected copper-containing ores and refined them into copper with higher purity through a series of complex processes. Then, according to their needs, craftsmen in the Han Dynasty would add a certain proportion of other metal elements, such as tin, to copper to form a bronze alloy with specific properties. This alloy had the advantages of high hardness, excellent toughness, and corrosion resistance, and was very suitable for making weapons, such as arrowheads. After completing the ore smelting, the bronze material obtained could be used to cast arrowheads. Furthermore, the combination of scanning electron microscopy (SEM) and energy-dispersive spectroscopy (EDS) revealed the complex structure of the sample surface: the fully mineralized layer, intermediate transition layer, and original core structure were clearly layered. Among them, the fully mineralized layer was mainly composed of copper oxide (CuO) and chalcopyrite (Cu_2_O), and its thickness was about 20–30 microns according to SEM cross-section observation, showing the typical characteristics of long-term burial corrosion. This discovery provides key clues for understanding the chemical evolution of arrowheads in underground environments.
(2) Chemical composition analysis: The EDS analysis results show that the average content of copper (Cu) in most of the samples was between 87% and 89%, and the content of tin (Sn) was about 10%, which is consistent with the typical composition of ancient bronzes. It is worth noting that the content of lead (Pb) in sample 1 was abnormally high, reaching 11.19% (as verified with ICP-MS), far exceeding the lead content in conventional bronze alloys. Combining historical documents and the archeological background, researchers speculate that this may have been due to deliberate additions during the manufacturing process to enhance the hardness or toughness of the arrows, or element migration caused by the soil environment during burial. In addition, trace amounts of molybdenum (Mo) were detected in samples 4 and 8, with contents of 0.21% and 0.18%, respectively (confirmed with XRF). This finding suggests that the soil environment and human activities influenced the burial conditions of arrows. The diversity of these elemental compositions not only reflects the complexity of ancient manufacturing processes but also reveals the profound impact of environmental conditions on material properties.
(3) Casting process and corrosion behavior study: Through electrochemical impedance spectroscopy (EIS) and polarization curve testing, the researchers found that there were significant differences in the corrosion behavior between the arrow longitudinal section and the cross-section. The longitudinal section exhibits strong corrosion resistance due to its dense structure and orderly grain arrangement, and its corrosion rate was calculated to be about 0.01 mm/y. In contrast, the cross-section was more corroded due to an irregular grain arrangement and uneven stress distribution, with a corrosion rate of about 0.05 mm/y. This discovery emphasizes the profound impact of manufacturing processes on the material properties and long-term preservation status and provides a scientific basis for the protection and restoration of archeological relics.
(4) Historical value and technical level assessment: Through the comprehensive analysis of eight arrowhead samples, combined with X-ray diffraction (XRD) and thermogravimetric analysis (TGA) data, the researchers preliminarily inferred that these arrowheads originated from the large-scale standardized production system of bronze weapons in the Minyue Kingdom. The microstructure and chemical compositions of samples 2, 3, and 6 were highly consistent, and principal component analysis (PCA) further confirmed that they may have been from the same batch, which confirms the superb organizational ability and technical level of the Minyue Kingdom in bronze weapon manufacturing. Combined with historical documents, this study not only shows that the Minyue Kingdom demonstrated outstanding skills in the innovation and optimization of bronze manufacturing technology in the Han Dynasty, such as the use of unique alloy ratios and casting techniques, but also reflects the complex social, economic, and military background at that time.

### 5.2. Research Limitations and Future Perspectives

Although this study reveals the material properties and casting process of bronze arrowheads in the Minyue Kingdom through multiple technical means, there are still some shortcomings. (1) The number of samples was limited, covering only eight arrowheads unearthed from the East City Gate warehouse, which made it difficult to fully reflect the diversity of bronze arrowheads in the site. (2) Although high-precision analysis methods, such as ICP-MS and XRF, were used in this study, sufficient evidence has not been provided for the source and function of special elements (such as Pb and Mo), and further in-depth research using isotope analysis or micro-Raman spectroscopy is needed. (3) Although the literature comparison study provides valuable historical background, there is a lack of systematic comparison with bronze weapons in other regions, such as through cluster analysis and factor analysis, which limits the in-depth understanding of the regional characteristics of the Minyue Kingdom’s casting technology and the cultural exchange path.

Future research could delve deeper into the following areas: (1) the samples could be expanded to include arrowheads from different excavation sites within the site, and high-throughput sequencing and big data analysis methods could be used to explore the changes in technology through time and space. (2) High-precision characterization techniques, such as transmission electron microscopy (TEM), atomic force microscopy (AFM), and simulation experiments, could be used to accurately identify the key steps in arrow production and their impact on material properties. (3) The comparative study of bronze weapons with other regions could be deepened, and the transmission path and influence range of the Minyue Kingdom’s cultural technology could be revealed by constructing network maps and using spatial analysis techniques. These studies will not only deepen our understanding of the Minyue Kingdom’s bronze arrowheads but will also strengthen the scientific basis for the research and protection of ancient bronzes.

## Figures and Tables

**Table 1:** Basic information of 8 samples.

Sample	Sample Site	Corrosion Status	Sectional Direction
1	Yuanbao Hill Smeltery and Residence Site	Moderate corrosion	Cross-section
2	Huang’gua Hill Smeltery and Residence Site	Moderate corrosion	Longitudinal section
3	South Gate Site	Slight corrosion	Cross-section
4	East Gate Site	Slight corrosion	Cross-section
5	Zhaocuo Dyke Smeltery and Residence Site	Moderate corrosion	Cross-section
6	Fulin Hill Smeltery and Residence Site	Moderate corrosion	Longitudinal section
8	North Gate Site	Slight corrosion	Longitudinal section
9	West Gate Site	Slight corrosion	Longitudinal section

Source: Author’s statistics.

**Table 2:** Metallographic observation results of these 8 bronze arrowhead samples.

Sample	50×	100×	200×	500×	Sectional Direction
1	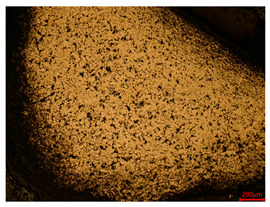	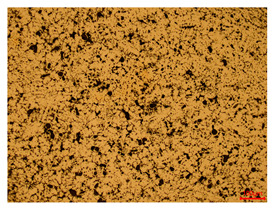	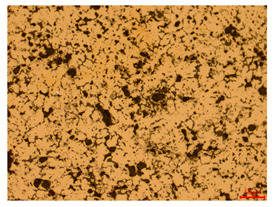	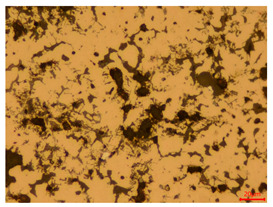	Cross-section
2	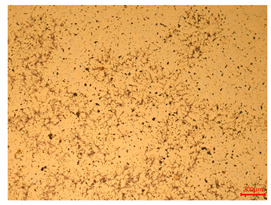	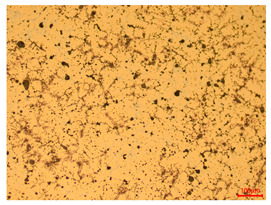	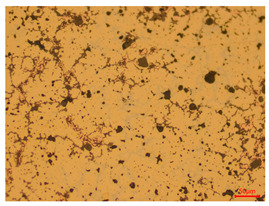	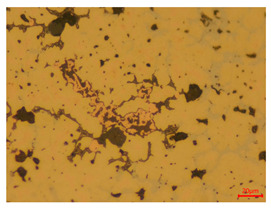	Longitudinal section
3	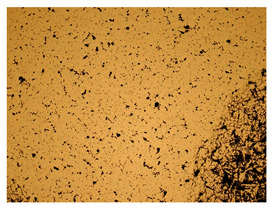	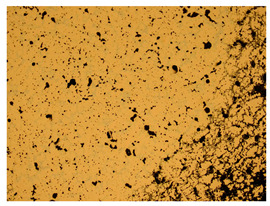	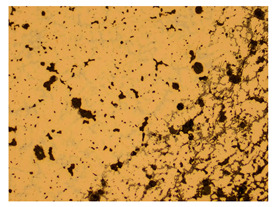	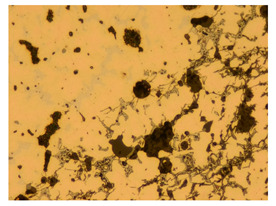	Cross-section
4	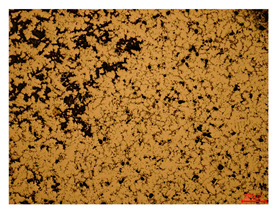	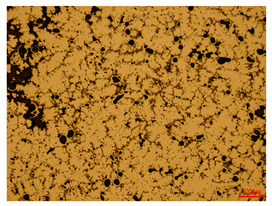	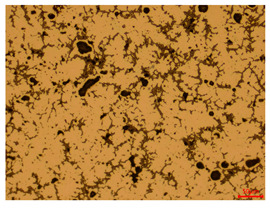	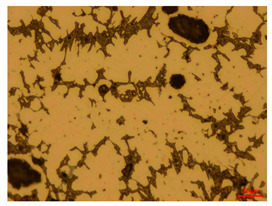	Cross-section
5	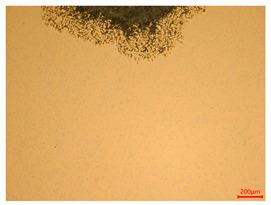	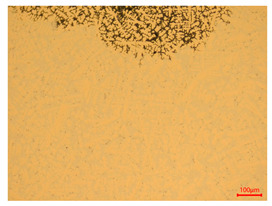	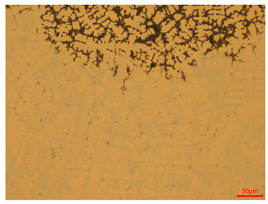	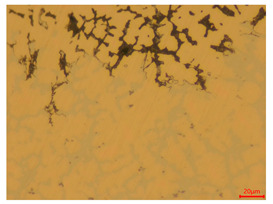	Cross-section
6	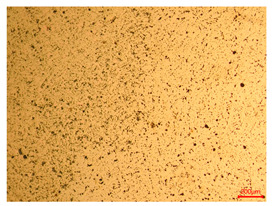	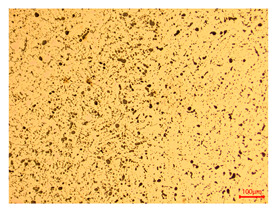	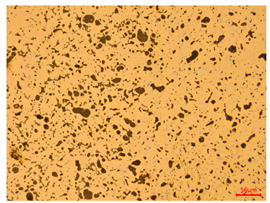	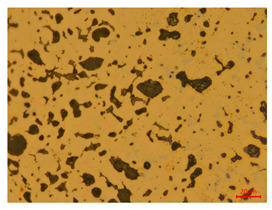	Longitudinal section
8	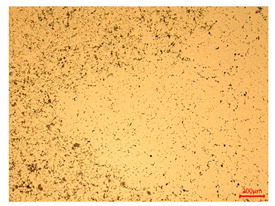	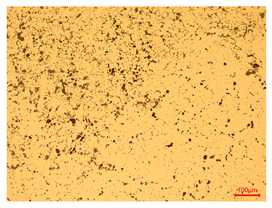	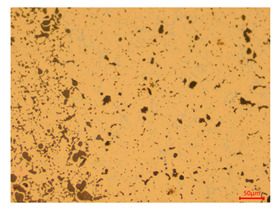	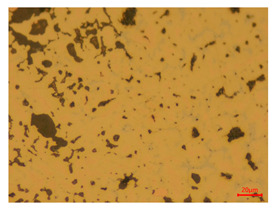	Longitudinal section
9	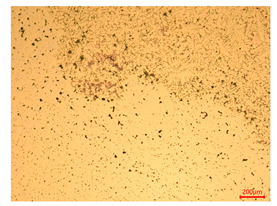	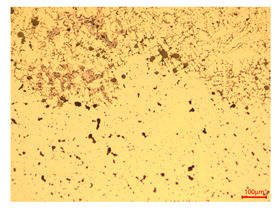	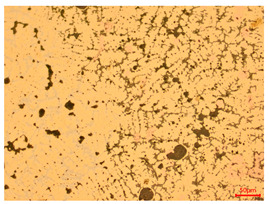	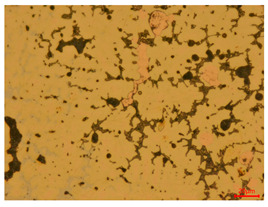	Longitudinal section

Note: The image at 50-times magnification is at a scale of 200 μm, the image at 100-times magnification is at a scale of 100 μm, the image at 250-times magnification is at a scale of 50 μm, and the image at 500-times magnification is at a scale of 20 μm.

**Table 3:** Analysis results of metallographic structure characteristics of 8 bronze arrowheads.

Sample	Sectional Direction	Microstructural Characteristics
1	Cross-section	The matrix comprises an α-phase segregation structure, with inclusions of varying sizes that are primarily present in a dot-like and irregular shape. These inclusions are uniformly distributed, and evident signs of corrosion traces are observable.
2	Longitudinal section	The matrix consists of fine α-dendritic segregation structures, with inclusions of varying sizes that primarily exhibit a punctuate and irregular morphology. These inclusions are uniformly distributed throughout the matrix, and distinct signs of corrosion traces are visible.
3	Cross-section	The matrix consists of fine α-dendritic segregation structures, with inclusions of varying sizes that primarily exhibit a punctuate and irregular morphology. These inclusions are uniformly distributed throughout the matrix, and distinct signs of corrosion traces are visible.
4	Cross-section	The matrix is characterized by a fine α-dendritic segregation structure, with inclusions of varying sizes that predominantly appear in a punctate and irregular shape. These inclusions are relatively uniformly distributed, and prominent signs of corrosion traces are observable.
5	Cross-section	The matrix consists of a fine α-dendritic segregation structure, with inclusions uniformly distributed in a punctate pattern, and evident signs of corrosion traces are observable.
6	Longitudinal section	The matrix comprises a fine α-dendritic segregation structure, with inclusions of varying sizes that primarily exhibit a punctuate and irregular morphology. A notable layering phenomenon is present within the structure.
8	Longitudinal section	The matrix is characterized by a fine α-dendritic segregation structure, within which inclusions of varying sizes are primarily present in a punctuate and irregular shape. A distinct layering phenomenon is evident, and prominent signs of corrosion traces are observable.
9	Longitudinal section	The matrix consists of a fine α-dendritic segregation structure, with inclusions of varying sizes that primarily exhibit a punctuate and irregular morphology and are relatively uniformly distributed.

Source: Authors’ statistics.

**Table 4:** Element overlay distribution of 8 bronze arrowhead samples.

Sample	Image	Element Overlay
1	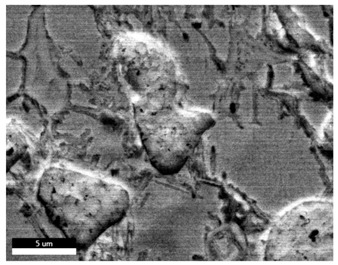	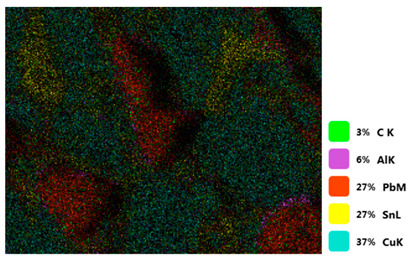
2	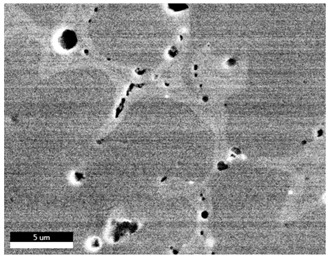	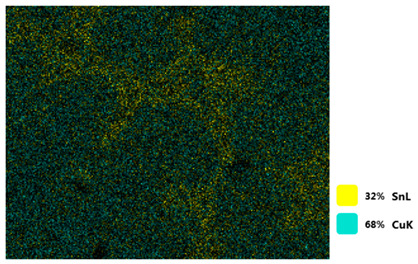
3	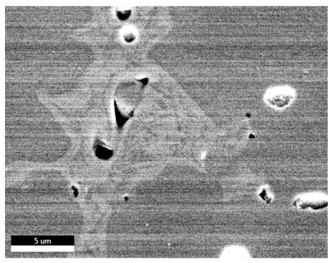	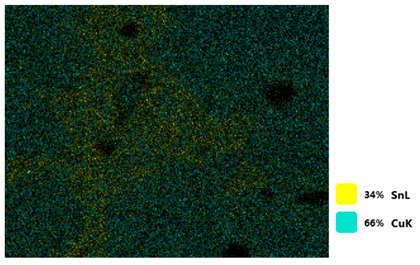
4	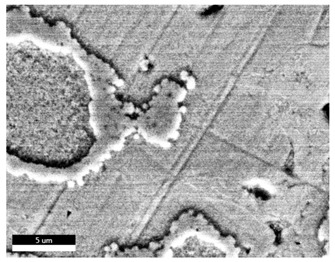	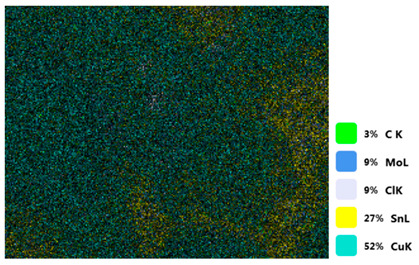
5	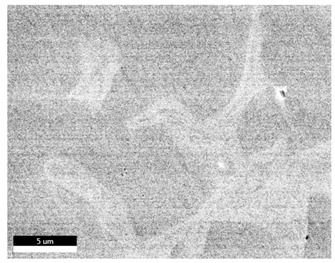	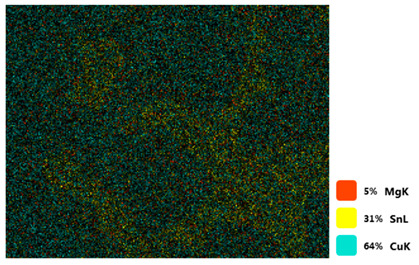
6	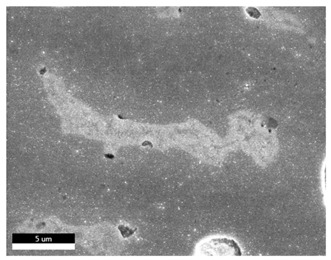	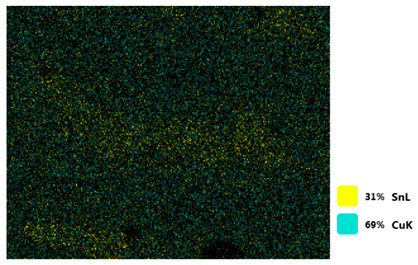
8	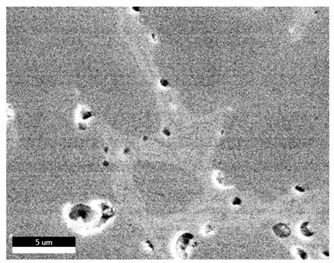	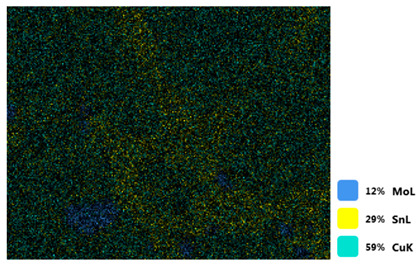
9	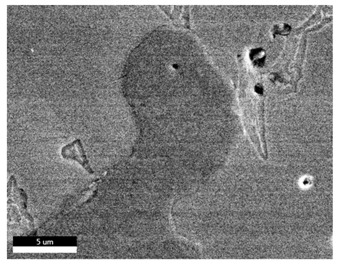	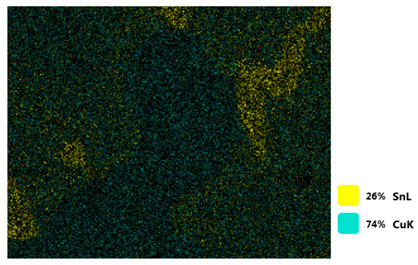

Source: Authors’ statistics.

**Table 5:** Element distribution of 8 bronze arrowhead samples.

No.	Element Distribution							
	Carbon (C)	Aluminum (Al)	Lead (Pb)	Tin (Sn)	Copper (Cu)	Molybdenum (Mo)	Chlorine (Cl)	Magnesium (Mg)
	Energy Levels (K Shell)	Energy Levels (K Shell)	Energy Levels (M Shell)	Energy Levels (L Shell)	Energy Levels (K Shell)	Energy Levels (L Shell)	Energy Levels (K Shell)	Energy Levels (K Shell)
1	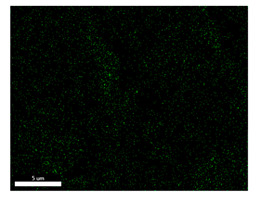	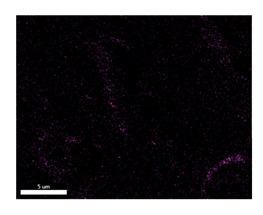	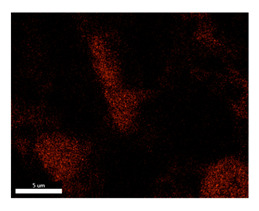	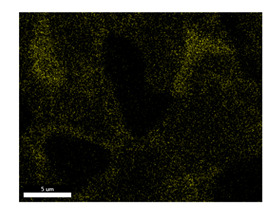	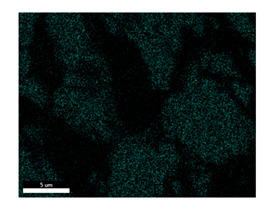	/	/	/
2	/	/	/	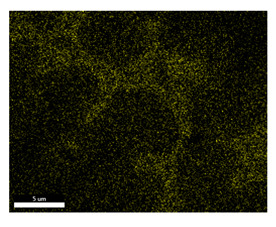	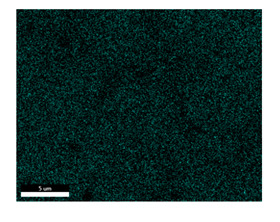	/	/	/
3	/	/	/	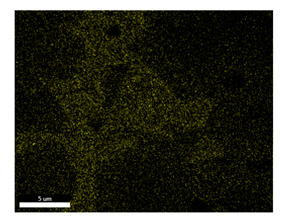	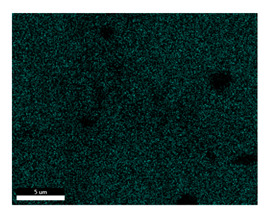	/	/	/
4	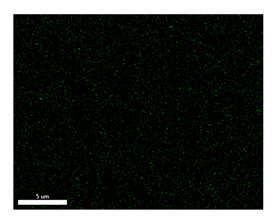	/	/	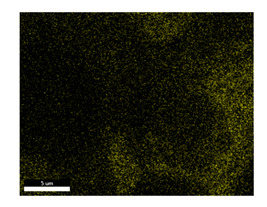	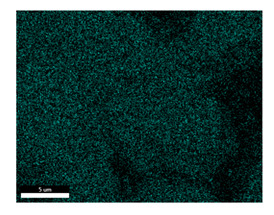	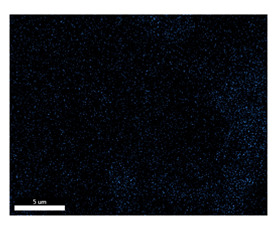	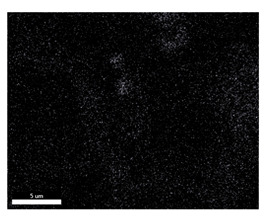	/
5	/	/	/	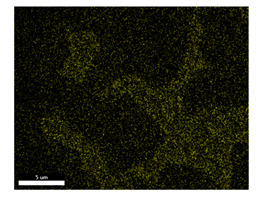	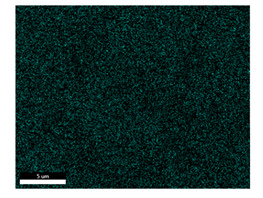	/	/	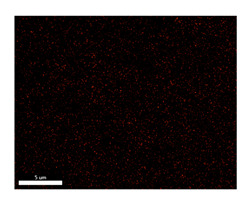
6	/	/	/	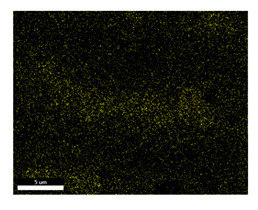	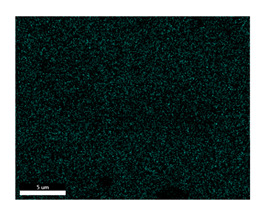	/	/	/
8	/	/	/	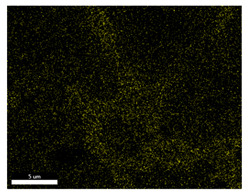	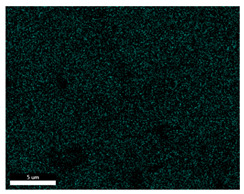	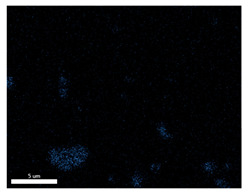	/	/
9	/	/	/	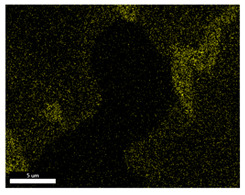	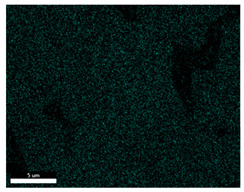	/	/	/

Source: Authors’ statistics.

**Table 6:** Energy-dispersive spectrometer composition analysis results for 8 bronze arrowhead samples.

Sample	Sample Site	Sectional Direction	Primary Components wt%				
			Cu	Sn	Pb	C	ELSE
1	Yuanbao Hill Smeltery and Residence Site	Cross-section	68.75	15.54	11.19	4.08	Al: 0.45
2	Huang’gua Hill Smeltery and Residence Site	Longitudinal section	87.79	12.21	-	-	-
3	South Gate Site	Cross-section	87.02	12.98	-	-	-
4	East Gate Site	Cross-section	82.77	12.87	-	3.04	Mo: 0.79, Cl: 0.53
5	Zhaocuo Dyke Smeltery and Residence Site	Cross-section	86.96	12.12	-	-	Mg: 0.92
6	Fulin Hill Smeltery and Residence Site	Longitudinal section	88.79	11.21	-	-	-
8	North Gate Site	Longitudinal section	86.00	12.50	-	-	Mo: 1.50
9	West Gate Site	Longitudinal section	91.34	8.66	-	-	-

Source: Authors’ statistics.

**Table 7:** eZAF Smart Quant results.

Sample	Element	Energy Levels	Weight (%)	Atomic (%)	Error (%)
1	Carbon (C)	K shell	4.08	20.91	10.78
	Aluminum (Al)	K shell	0.45	1.02	16.75
	Lead (Pb)	M shell	11.19	3.33	2.82
	Tin (Sn)	L shell	15.54	8.07	3.33
	Copper (Cu)	K shell	68.75	66.67	3.72
2	Tin (Sn)	L shell	12.21	6.93	3.34
	Copper (Cu)	K shell	87.79	93.07	3.45
3	Tin (Sn)	L shell	12.98	7.40	3.33
	Copper (Cu)	K shell	87.02	92.60	3.45
4	Carbon (C)	K shell	3.04	15.00	11.07
	Molybdenum (Mo)	L shell	0.79	0.49	7.72
	Chlorine (Cl)	K shell	0.53	0.88	8.12
	Tin (Sn)	L shell	12.87	6.42	3.23
	Copper (Cu)	K shell	82.77	77.20	3.45
5	Magnesium (Mg)	K shell	0.92	2.50	13.49
	Tin (Sn)	L shell	12.12	6.77	3.72
	Copper (Cu)	K shell	86.96	90.73	3.46
6	Tin (Sn)	L shell	11.21	6.33	4.46
	Copper (Cu)	K shell	88.79	93.67	3.59
8	Molybdenum (Mo)	L shell	1.50	1.06	6.90
	Tin (Sn)	L shell	12.50	7.14	4.03
	Copper (Cu)	K shell	86.00	91.80	3.60
9	Tin (Sn)	L shell	8.66	4.83	4.75
	Copper (Cu)	K shell	91.34	95.17	3.45

Note: The Errors (%) heading in Table 7 refers to relative error ranges estimated based on comprehensive factors, such as the EDS signal intensity, background noise, instrument performance, and algorithm correction. Its role is to indicate the credibility and uncertainty of the data, thereby helping researchers to better judge which elements the main components of the sample are, and which may be noise caused by environmental influences or analytical errors.

**Table 8:** Repair and protection methods for bronze arrowheads.

Measures	Method	Specific Practices
Mechanical cleaning	Low-speed polishing	Applied a low-speed metallographic polisher (QATM Saphir 560) and 4000-grit sandpaper to gently remove the surface mineralization and floating rust. For sample 1, particular care was taken to preserve the dendrite structure where Pb was concentrated.
	Controlled air abrasion	Conducted air abrasion using alumina powder under low pressure for samples 4 and 8 to remove compacted soil particles without damaging the surface corrosion layers.
Chemical cleaning	EDTA solution	Diluted EDTA (5%) with ultrasonic oscillation was used on samples 6 and 9 to dissolve the oxide layers. For localized severe corrosion (sample 1), a 1% benzotriazole (BTA) solution was applied to inhibit the further oxidation of the Cu–Pb compounds.
	pH-controlled rinsing	Used deionized water adjusted to pH 7 for all the samples to rinse and neutralize residues from soil deposits, preventing secondary reactions.
Stabilization treatment	Freeze-drying	Freeze-drying at −50 °C was applied to remove adsorbed water from samples 2 and 3. Ion-exchange resin was used to reduce the CuCl_2_ content in samples where malachite formation was evident.
	Neutral sealer coating	Applied Paraloid B-72 to samples 5 and 6 after chemical cleaning to form a moisture-proof protective layer. This coating was particularly effective in stabilizing microcracks in high-tin regions.
Controlled storage environment	Humidity control	Samples were stored at 40% relative humidity to prevent Cu_2_Cl_2_’s conversion into malachite under high-humidity conditions. For sample 8, desiccants were placed to stabilize microstructures identified through SEM.
	Temperature control	Maintained storage temperatures between 18 °C and 22 °C to avoid intergranular crack propagation in samples with detected thermal stress (e.g., sample 4).
	Low-oxygen atmosphere	Sample 4 was placed in a sealed nitrogen atmosphere (<5% oxygen) to suppress oxidation. Sulfur dioxide was introduced to simulate the stabilization of molybdenum detected in the sample.
Corrosion inhibitor application	Immersion in BTA solution	Samples with active corrosion (e.g., samples 1 and 8) were locally immersed in a 2% BTA solution for 24 h to enhance the passivation layer and reduce corrosion progression.
	VCI application	Placed volatile corrosion inhibitors (VCIs) in storage spaces containing samples 5 and 6 to inhibit atmospheric corrosion. The inhibitors were selected based on the Cu–Sn alloy stabilization requirements.

Source: Authors’ statistics.

## Data Availability

The original contributions presented in this study are included in the article. Further inquiries can be directed to the corresponding author.
